# Interactive, Personalized Patient Decision Aid for COVID-19 Vaccination in Canada: User-Centered Design Approach

**DOI:** 10.2196/86283

**Published:** 2026-04-16

**Authors:** Doriane Etienne, Patrick M Archambault, Isaac I Bogoch, Christine T Chambers, Andrea D Chittle, Juliette Demers, S Michelle Driedger, Ève Dubé, Marie-Pierre Gagnon, Teresa Gavaruzzi, Anik Giguère, Nathalie Grandvaux, Kelly Grindrod, Hina Hakim, Samira Jeimy, Jason Kindrachuk, Annie LeBlanc, Shannon E MacDonald, Ruth Ndjaboue, Magniol Noubi, Rita Orji, Jean-Sébastien Paquette, Elizabeth Parent, Jean-Sébastien Renaud, Beate Sander, Monica Taljaard, Dana Tannenbaum Greenberg, Marie-Claude Tremblay, Sabina Vohra-Miller, Vivian A Welch, Holly O Witteman

**Affiliations:** 1 Department of Family and Emergency Medicine, Faculty of Medicine, Laval University, Québec City, QC, Canada; 2 VITAM – Centre de recherche en santé durable, Université Laval, 2480 Chemin de la Canardière, Québec City, QC, G1J 2G1, Canada, 1 418 663-5313 ext 12286; 3 Research Centre of the CHU de Québec, Laval University, Québec City, QC, Canada; 4 Centre de Recherche Intégrée Pour un Système Apprenant en Santé et Services Sociaux du Centre Intégré de Santé et de Services Sociaux de Chaudière-Appalaches, Lévis, QC, Canada; 5 Divisions of General Internal Medicine & Infectious Diseases, Toronto General Hospital, University Health Network, Toronto, ON, Canada; 6 Departments of Psychology and Neuroscience & Pediatrics, Faculties of Science and Medicine, Dalhousie University, Halifax, NS, Canada; 7 Centre for Pediatric Pain Research, IWK Health, Halifax, NS, Canada; 8 Department of Family Medicine, McMaster University, Hamilton, ON, Canada; 9 Department of Biochemistry, Faculty of Science, McGill University, Montréal, QC, Canada; 10 College of Community and Global Health, Rady Faculty of Health Sciences, University of Manitoba, Winnipeg, MB, Canada; 11 Faculty of Nursing Sciences, Laval University, Québec, QC, Canada; 12 Department of Medical and Surgical Sciences, University of Bologna, Bologna, Italy; 13 Department of Biochemistry and Molecular Medicine, Faculty of Medicine, Université de Montréal, Montréal, QC, Canada; 14 Centre de Recherche du Centre Hospitalier de l'Université de Montréal, Montréal, QC, Canada; 15 School of Pharmacy, Faculty of Science, University of Waterloo, Waterloo, ON, Canada; 16 Division of Clinical Immunology and Allergy, Department of Medicine, Western University, London, ON, Canada; 17 Department of Medical Microbiology & Infectious Diseases, Rady Faculty of Health Sciences, University of Manitoba, Winnipeg, MB, Canada; 18 Faculty of Nursing, University of Alberta, Edmonton, AB, Canada; 19 Faculty of Letter and Human Sciences, School of Social Work, Université de Sherbrooke, Sherbrooke, QC, Canada; 20 Centre de Recherche sur le Vieillissement, Sherbrooke, QC, Canada; 21 Faculty of Computer Science, Dalhousie University, Halifax, NS, Canada; 22 Health Systems and Policy Research Collaborative Centre, University Health Network, Toronto, ON, Canada; 23 Methodological and Implementation Research, Ottawa Hospital Research Institute, Ottawa, ON, Canada; 24 Diabetes Action Canada, Toronto, ON, Canada; 25 Dalla Lana School of Public Health, Division of Clinical Public Health, University of Toronto, Toronto, ON, Canada; 26 Bruyere Health Research Institute, Ottawa, ON, Canada

**Keywords:** COVID-19 vaccination, patient decision aid, web-based tool, iterative human-centered design, digital health, health literacy, user engagement, public health communication, data visualization, personalized avatar

## Abstract

**Background:**

The COVID-19 pandemic highlighted the need for practical digital health tools to support informed decision-making amid rapidly evolving evidence and widespread misinformation.

**Objective:**

We iteratively developed and refined VaxDA-C19, a bilingual (English and French) web-based patient decision aid designed to support informed decision-making in Canada about COVID-19 vaccination. VaxDA-C19 integrates interactive and personalized features aimed to enhance vaccine confidence, reduce cognitive overload, and respond to diverse informational needs.

**Methods:**

VaxDA-C19 was developed using an iterative, user-centered design approach. Throughout the development process, we involved a citizen panel, health care professionals, user experience designers, and scientific experts to guide refinements. We also conducted usability testing sessions with adults in Canada, using semistructured interviews, comparative testing, and think-aloud protocols with thematic analysis. We ultimately conducted 4 design cycles in total with adults in Canada (users) and expert reviewers (experts). Cycle 1 involved 9 people (9 users), cycle 2 involved 22 people (22 users), cycle 3 involved 9 people (3 users and 6 experts), and cycle 4 involved 9 people (9 experts).

**Results:**

In cycle 1, user feedback guided design decisions about how to present quantitative information and technical vaccine descriptions more simply. In cycle 2, while most users (9/11, 82%) favored in-depth explanations of vaccine development, a few raised concerns about content that could be perceived as politically charged. Cycle 3 identified usability improvements, including more explicit navigation controls, simplified medical terminology, and optimized interactive components (avatars and sliders). Expert reviews in cycle 4 refined linguistic consistency, mobile responsiveness, content transparency, and scientific accuracy, emphasizing explicit instructional guidance and bilingual accessibility.

**Conclusions:**

Our iterative process produced a personalized, bilingual digital decision aid to support evidence-informed, values-congruent decisions about COVID-19 vaccination. A randomized controlled trial will further evaluate VaxDA-C19’s impact on vaccination intentions, knowledge retention, emotional responses, decisional conflict, and decisional regret. If it proves effective, the patient decision aid may also be used as a platform to support other vaccine decisions, namely, influenza, measles, shingles, pertussis, and potentially other emerging infectious diseases.

## Introduction

The COVID-19 pandemic exposed challenges in public health decision-making driven by rapidly evolving scientific evidence and widespread misinformation [1-8]. People had to make complex choices, such as whether to accept vaccination or adopt preventive behaviors, often under conditions of uncertainty [4,9-13]. These challenges highlighted the need for decision support tools that empower users to make values‐congruent choices informed by the best available evidence [1,10,14-21].

Patient decision aids support shared decision-making by making decisions explicit, providing balanced and evidence-based information about options, and helping individuals clarify their values [22]. Robust evidence from meta-analyses and systematic reviews, including a comprehensive Cochrane review, demonstrates that patient decision aids significantly improve knowledge, accuracy of risk perceptions, and alignment between decisions and personal values, while also reducing decisional conflict   [23,24]. In the context of vaccination, a systematic review and meta-analysis of 5 randomized controlled trials (total n=2158) found that patient decision aids for vaccine decisions had significant positive effects on both vaccine intentions (odds ratio 1.89, 95% CI 1.20‐2.97) and uptake (odds ratio 1.77, 95% CI 1.25‐2.52) [25].

Emerging evidence suggests that interactive and personalized digital tools can encourage behavior change among individuals who have not yet engaged in recommended practices, reinforce existing behaviors, and provide reassurance [26-29]. Such multiple purposes may be particularly critical during periods of crisis, when individuals seek validation for their actions and guidance for future decisions [30,31].

As COVID-19 vaccines move toward regular annual or biannual vaccines in Canada, similar to influenza vaccines, there is a need for patient decision aids that can be easily updated to reflect updated vaccines and recommendations. To this end, we created VaxDA-C19, a web-based vaccine (Vax) patient decision aid (DA) designed to support COVID-19 (-C19) vaccination decisions in Canada. VaxDA-C19 is designed to support multiple languages and to provide information in a layered way, meaning that people who want to quickly access basic information can view brief bullet points, while those who want to know more can expand sections, get more details, and access original references. Our goals were to provide clear, evidence-based information on vaccination options, benefits, and risks to oneself and others while reducing cognitive overload. This paper describes VaxDA-C19’s design and development process.

## Methods

### Overview and Theoretical Frameworks

We developed VaxDA-C19 using an iterative, user-centered design approach. Our overall design process followed the International Patient Decision Aids Standards [32]. The patient decision aid was structured according to the Ottawa Decision Support Framework [33]. The web-based design and development approach combined human-centered design principles [34] and a Scrum framework [35]. Human-centered design was implemented via early and ongoing involvement of users and stakeholders, iterative prototyping, and cycle-by-cycle usability feedback to enhance content layout, navigation, and overall user experience (UX) [34]. Scrum served as a structure for delivering small, incremental updates through brief development cycles, with backlog prioritization and periodic review sessions to ensure that implementation aligned with new evidence and user input [35]. We tailored Scrum for this project by synchronizing review points with usability testing rounds. These rounds included checkpoints to validate content and evidence for modifications to vaccine information or recommendations. We also used streamlined remote meetings to accommodate the team and respond to evolving guidelines.

Throughout the development process, we involved a citizen panel, health care professionals, UX designers, and scientific experts to guide refinements. We also conducted usability testing sessions with adults in Canada, using semistructured interviews, comparative testing, and think-aloud protocols with thematic analysis. We ultimately conducted 4 design cycles in total with adults in Canada and expert reviewers. Cycle 1 involved 9 people (all prospective users), cycle 2 involved 22 people (all prospective users), cycle 3 involved 9 people (3 prospective users and 6 experts), and cycle 4 involved 9 people (9 experts).

### Citizen Panel

To help guide the overall project, a citizen panel of 6 individuals, comprising people from a range of age groups, languages, educational backgrounds, health statuses, and geographic regions (Alberta, Saskatchewan, Ontario, and Québec) provided feedback about the design and development of the patient decision aid and other interventions (reported separately) as well as the user testing methods and results. In the context of COVID-19 restrictions in place at the beginning of the project, the citizen panel was assembled from existing contacts with patient and citizen partners across a range of prior projects and life circumstances. This group met by Zoom (Zoom Video Communications) for 2 hours at a time to discuss ongoing project design, development, and study planning. This group served as an advisory panel, distinct from user testing with previously unknown members of the general public, described below [34].

### Expert Involvement

Health care professionals and research experts, including infectious disease researchers, epidemiologists, primary care providers, and 2 UX designers (internal and external experts), participated in the iterative process as expert reviewers. The designers’ expertise covered interaction design and usability evaluation (UX research and testing) as well as visual and information design. The internal designer was part of the senior author’s (HOW) human-centered digital health research team and supported participatory design activities, including planning and conducting user testing in field settings or in a human-computer interaction laboratory. The external designer is a UX lead specializing in design systems, including the development and evolution of interface components. Depending on their exact area of expertise, the designers’ roles were to support content presentation, interface coherence, and usability of the web-based patient decision aid [34].

### Ethical Considerations

This study received ethics approval from the “Comité d’éthique de la Recherche en Sciences de la Santé” of the Université Laval (approval: 2020‐222). All participants received detailed study information and provided written informed consent before participation. We offered user testing participants US $28.80 for their time and to help defray internet costs. We removed all identifying details of participants from reports and ensured the confidentiality of data according to institutional policies.

### Recruitment for User Testing

In light of COVID-19 restrictions in place during the bulk of this project, we used online tools to conduct our user tests. Specifically, we recruited user testing participants through tailored Facebook advertisements, then conducted user testing sessions using Zoom, with participants sharing their screen with the interviewer. In line with the objectives of human-centered design, the recruitment process involved a nonprobability convenience sample to support rapid, iterative refinement. The findings were intended to inform design improvements rather than statistical generalization [36]. Eligible participants for user testing were adults (≥18 years) residing in Canada, able to communicate in English or French, and possessing basic computer skills. Individuals without internet access or unable to provide informed consent were excluded. We therefore selected Facebook options to advertise to people who were 18 years and older across Canada and who indicated an education level of either an associate degree (eg, 2-year postsecondary diploma), high school graduate, or unspecified. We did not exclude people who had other educational statuses but used tailored recruitment in an effort to include people across a range of backgrounds, as our previous experiences suggested that people who respond to such advertisements are often more highly educated. We stopped recruiting for each cycle after reaching thematic saturation in comments and usability issues identified.

### Overall Design of the Intervention

We used Figma design software to iteratively design the overall structure and appearance of VaxDA-C19. As described further in the Results section, the patient decision aid ultimately included an introductory section in which the patient decision aid is presented, and the user selects the age group of the person for whom a decision is required (themself or their child), their province or territory of residence (which determines which vaccines are available), and the decision they are making (whether or not to get a primary series of vaccines, whether or not to get a booster dose, which vaccine to get, or which booster to get). The user is then invited to create an avatar representing themself or their child. This avatar is used in the next section, which describes the individual-level benefits and risks of the vaccine. VaxDA-C19 offers layered, straightforward summaries of evidence tailored to the user’s selected age, group, province or territory, and decision, including vaccine availability and eligibility based on current authorizations and guidelines. It presents individual benefits and harms in both text and visual risk formats, with links to public health sources and peer-reviewed evidence. The top of the page provides a summary, and the user can click to view more detailed explanations below the summary. On the next page, the user can choose to create up to 8 more avatars representing people around them or their child or to have additional avatars auto-generated. They may then view or skip a brief (2.5-minute) animation showing how vaccination may protect those around us [37]. This optional brief animation explains community immunity and can be viewed or skipped. At the same time, core evidence on individual risks and benefits, along with the rest of the patient decision aid, remains accessible at all times. Following this optional component, the user is invited to reflect on what matters to them, relevant to the decision, using a values clarification method previously developed and tested in the context of treatment decisions and influenza vaccination decisions [38]. Finally, the user has the option to read answers to frequently asked questions (FAQs) and to learn more about our team. A short walk-through video is available on YouTube [39].

### Design and Development Approach

**Figure 1.**
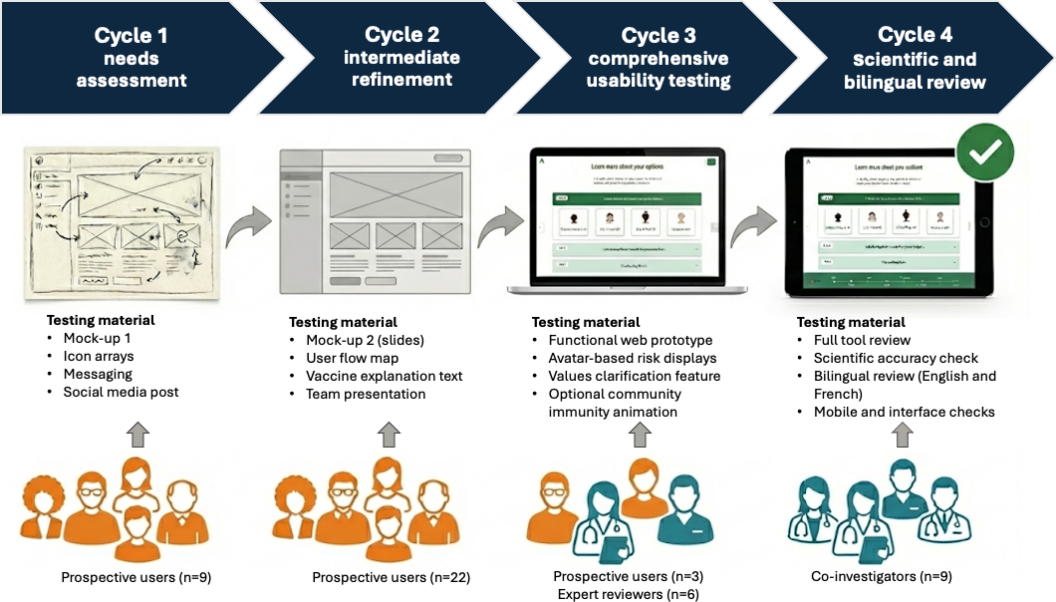
Iterative development cycles of VaxDA-C19, a web-based patient decision aid.

Our multidisciplinary team followed a human-centered design over 4 iterative cycles, summarized in Figure 1 [34]. We reported our patient decision aid development process using the DEVELOPTOOLS reporting checklist, provided in Checklist 1 [40]. As per the International Patient Decision Aids Standards [32], our design process was structured in multiple testing cycles, each involving small to medium user samples (3‐31 participants, no overlap between cycles). This aligns with cognitive ergonomics standards [41-43], which state that iterative testing with small samples efficiently identifies approximately 85% of usability issues per cycle. After each cycle, the team met to review findings and make decisions about modifications. The citizen panel met separately throughout the project to offer advice from their viewpoints.

### Data Collection and Analysis

We used Zoom to conduct user testing interviews. We evaluated whether VaxDA-C19 met its communication goals, and then applied a rapid thematic analysis [44,45] to qualitative data from interviews, comparative testing, and think-aloud sessions. Two trained research associates (EP and HH) independently coded transcripts, first extracting functional requirements (ie, needed features, content adjustments, and navigation flows), and then flagging any usability issues. We resolved disagreements through discussion with the lead author (DE) and principal investigator (HOW) until we reached a consensus. Finally, DE, EP, and HOW organized the agreed functional requirements and identified bugs into a clear, detailed plan to guide the development team. We reported the qualitative components in line with the SRQR (Standards for Reporting Qualitative Research) and provided the completed checklist in Checklist 2 [46]. We documented cycle-by-cycle decisions and changes to support transparency.

### Iterative Cycles

Across all cycles, we presented participants with increasingly higher-fidelity versions of VaxDA-C19 prototypes. We asked participants to summarize the information, identify points of confusion or unanswered questions, and react and respond to wording.

#### Cycle 1

For the needs assessment, we developed an initial content mock-up of VaxDA-C19 focused on primary COVID-19 vaccination (first-dose decision). We based the mock-up’s content on contemporaneous recommendations from the Canadian National Advisory Committee on Immunization, the Institut National de Santé Publique du Québec, and Health Canada authorizations. Feedback from the citizen panel and content experts addressed clarity, scientific accuracy, and relevance. We then recruited participants (n=9) via tailored Facebook advertisements and conducted individual semistructured Zoom interviews, whose interview guides are shown in full in MultimediaAppendices 1  2. In each session, we asked participants to interpret icon arrays, react to hypothetical provider messaging, and consider a social media post.

**Figure 2.**
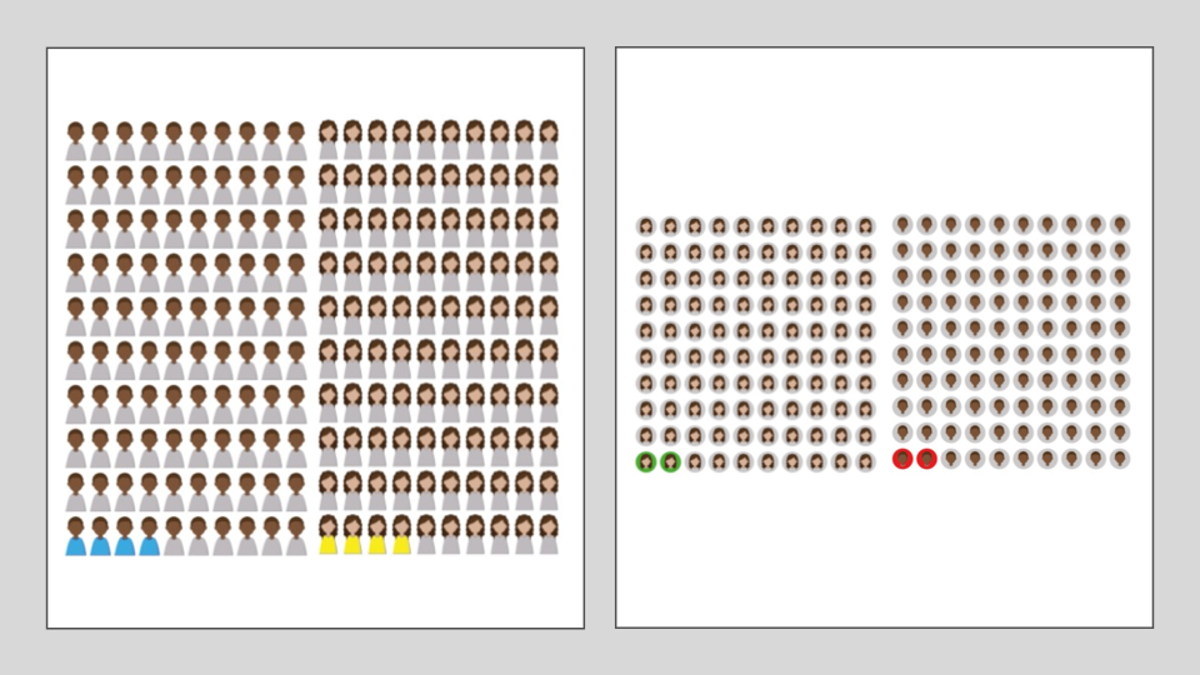
Icon arrays used during the first cycle.

For the icon arrays, as shown in Figure 2, we offered 2 icon array styles for risk communication: avatars with colored shirts and avatars with circular backdrops, and asked participants to compare these, focusing on how well or poorly these visual aids conveyed risk data.

For the provider messaging, we asked people who were able to receive messages from their primary care provider,

Imagine your primary care health professional (e.g., family doctor, nurse practitioner) sends you a message like, “I got my COVID-19 vaccine when I was eligible. Now that you are eligible, I’d like to support you by answering any questions you might have. Here’s a link with some information: link. Please send me a message if you have more questions.” How would that make you feel? How might you react or respond?

We asked participants who were unable to receive messages to respond to a similar message, but imagining it was during an in-person visit. Full details are shown in Multimedia Appendix 1.

The proposed social media post aimed to explain why COVID-19 vaccines moved so rapidly through development, for use in a potential image-based post, such as an Instagram carousel. The proposed English text is present in Textbox 1. Full text is available in Multimedia Appendix 2.

Textbox 1. **English content for the social media intervention (Instagram carousel) explaining the accelerated development of COVID-19 vaccines.**

- 9 women can’t make a baby in 1 month, no matter how well they work together!
- But when a bunch of the world’s best scientists work together, with enough research funding, motivated by a pandemic that is disrupting lives around the world, vaccine development can happen faster than usual.
- Making a new vaccine usually involves months or years of waiting time. Waiting to get more research funding. Waiting for enough people to sign up for studies that make sure the vaccines are safe and work well. Waiting for approvals from independent regulatory authorities before starting to produce the vaccine for distribution.
- Usually, this waiting happens because the need for the vaccine isn’t considered urgent enough for everyone to drop everything else and make the new vaccine everyone’s top priority. But COVID-19 has been a different story. It has been a priority around the world.
- To make COVID-19 vaccines, they cut out a lot of the waiting. But they didn’t cut corners. All the safety checks are still being done. When COVID-19 vaccines are approved by Health Canada (Canada’s independent agency, staffed by top-notch scientists and not affiliated to any political party) you can trust that they are safe and they work.
- We are Canadian scientists, doctors, nurses, and experts in health and vaccines. None of us works for pharmaceutical companies. We don’t accept pharmaceutical funds either. We will get the COVID-19 vaccines for ourselves as soon as they are approved by Health Canada and available to us. Once they have been tested and approved for children, our children will get the vaccines, too.
- Still have questions about COVID-19 vaccines and don’t know where to ask? We want to use our scientific training to help answer your questions. Send us your question here. We will do our very best to answer as many as we can.

#### Cycle 2

**Figure 3.**
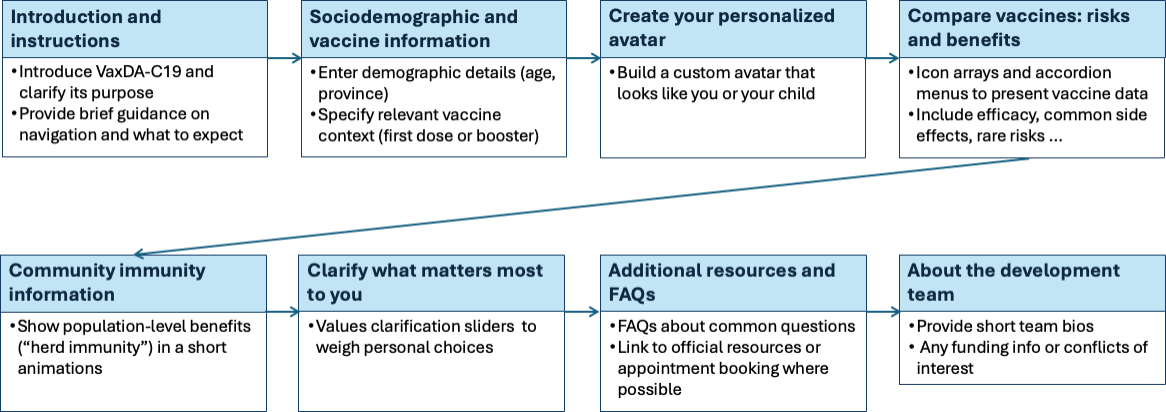
User flow representation of our web-based patient decision aid, VaxDA-C19. FAQ: frequently asked question.

Based on results from cycle 1, after identifying initial user needs, we developed the first version of content using Google Slides to create mock-ups of a proposed website. These mock-ups served as early prototypes, allowing us to conceptualize the user interface and gather feedback from the citizen panel on design, usability, and information presentation prior to investing substantial time in web development. Figure 3 maps the user flow. It began with a page of welcome text and instructions, then a page in which people indicate the age and province of residence of the person for whom a vaccine is being considered, then a page in which people build an avatar representing that person, then a page showing the individual-level risks and benefits of the vaccine for that person (using the avatar previously created to illustrate statistics), then a section showing the population-level benefits of vaccination, then a values clarification method to help people align their decision with what matters to them, and finally, FAQs, information about where to obtain a vaccine, references supporting the content, and information about our team.

We then recruited new participants (n=22) via tailored Facebook advertisements and conducted a second round of individual semistructured interviews on Zoom, again applying thematic analysis (MultimediaAppendices 3  4). In each interview, we asked: “In your own words, what was this text about?,” “Why do you think these vaccines arrived so quickly?,” and “What important questions about COVID-19 vaccines remain unanswered?” During these sessions, we used A/B testing to compare designs and content presentations of the team working on VaxDA-C19 and related projects. A/B testing, or split testing, involves presenting 2 versions (A and B) of a webpage or app to different user groups to determine which version performs better based on predefined metrics [47]. In this case, participants viewed 2 versions of the team presentation. Version A included only drawings, while version B included photographs and the names of research team members. Full details of the 2 designs are available in Multimedia Appendix 4.

#### Cycle 3

Cycle 3 moved VaxDA-C19 from static mock-ups into a fully functional web prototype, and then tested it with end users and experts. Based on results from cycle 2, we added 5 interactive features. These included having users create a personalized avatar to promote personalized user engagement [48,49] and to take advantage of the Proteus effect, a documented effect in which people identify with their avatar [50,51]. VaxDA-C19 then uses that personalized avatar in icon arrays to facilitate visual risk comparisons [52-54]. It also offers users the option to view a 2-minute visualization incorporating their avatar, previously developed by our team to illustrate how community immunity (herd immunity) may contribute to protection [48,49]. The visualization makes use of 4 theories or frameworks: the health belief model [55], Gestalt visual principles [56], the cognitive theory of multimedia learning [57,58], and the affect heuristic [59,60]. We also added a values clarification feature, as per International Patient Decision Aid Standards, to help users align their vaccine decision with their values [61,62]. Finally, we used accordion menus (menus that show a brief title and allow people to click to open for full information) to enable users to decide for themselves how much or how little information they wish to read about the available vaccines [63]. We built the prototype in Vue.js (HTML, CSS, and JavaScript) [64] with JSON-templated content. This architecture enabled rapid updates as new vaccine data and guidelines evolved.

Next, we conducted a third round of online user testing, again recruiting new participants (n=3) via tailored Facebook advertisements. Through individual semistructured interviews on Zoom, we used think-aloud protocols focused on multidevice compatibility, navigation, and overall usability (technical details in Multimedia Appendix 5).

Following user testing, the lead author (DE) presented the prototype at the 18th Biennial European Conference of the Society for Medical Decision-Making during the Society for Medical Decision-Making Core Course: Introduction to shared decision-making and patient decision aids. During the session, attendees engaged in a discussion in which attendees shared insights from their respective fields. As part of this discussion, the lead author (DE) had the opportunity to show the attendees the progress of our work and to get their feedback. This additional expert feedback (n=5) addressed some direction on medical content and interface navigation. In addition, an external UX designer (n=1) delivered targeted feedback after testing to complement the internal designer’s expertise.

While this cycle was underway, evolving public health guidelines suggested 4 distinct vaccination decisions: initial COVID-19 vaccination for those aged 12 years and older, booster doses for those aged 12 years and older, initial vaccination for children aged 6 months to 11 years, and booster doses for children aged 6 months to 11 years. We therefore restructured VaxDA-C19 accordingly.

#### Cycle 4

In cycle 4, we finalized VaxDA-C19, such that it might be ready for testing in an online randomized controlled trial. We updated its content to reflect the most recent Health Canada-authorized COVID-19 vaccines and national recommendations from the National Advisory Committee on Immunization. We then invited project coinvestigators to conduct an expert review; 9 experts provided detailed feedback. The expert reviewers were project coinvestigators with multidisciplinary expertise relevant to patient decision aids and COVID-19 vaccination, including digital health, human-centered design, virology, vaccinology, infectious disease epidemiology, primary care, public health, risk communication, health equity, and knowledge translation. Evaluation criteria encompassed scientific accuracy, linguistic clarity, content completeness, and user journey across all 4 vaccination decisions.

Based on this feedback, we prioritized key modifications, including removing irrelevant vaccine selection options for specific age groups, clarifying instructional text to specify required selections more clearly, incorporating contact information and funder logos on the homepage, and resolving layout inconsistencies, notably mobile interface challenges. Textbox 2 presents a synthesis of the key insights and considerations that emerged from our iterative design and development process.

Textbox 2. **Key points from the design and development process.**

Recruitment strategies: Recruitment through tailored social media advertisements (Facebook), complemented by internal dissemination via research team networks, effectively reached a diverse group of participants, including variations in educational level, age, language, and geographic location. Future studies could potentially benefit from additional strategies, such as direct collaboration with community-based organizations.

Iterative usability testing: Short, iterative usability cycles (4 cycles, sample sizes from 3 to 22 participants each) allowed rapid identification and correction of usability issues. Participants’ repeated feedback emphasizes the need to simplify interactive elements (eg, avatar creation, icon arrays, and value clarification sliders), which may have contributed to enhanced clarity and ease of use across testing cycles.

Flexible content management: Implementing JSON-based templates allowed efficient updates to rapidly evolving vaccine guidelines, easing the task of ensuring ongoing scientific accuracy. Early involvement of content experts contributed to the accuracy, clarity, and scientific rigor of the web-based patient decision aid.

Multidisciplinary input: Feedback from diverse interested parties (patients, health care professionals, user experience designers, and researchers) contributed a wide range of perspectives on the clinical relevance, usability, and accuracy of VaxDA-C19.

Table 1 provides an overview of each cycle, along with the major modifications we implemented.

**Table 1.** Cycle summaries.

Cycle	Objective	Sample size, n	Procedure used	Main modifications
1	Needs assessment	9 users	Semistructured interviews Thematic analysis Comparative testing	Clarified initial content
2	Intermediate refinement	22 users	Google Slides mock-ups Comparative testing Thematic analysis	Added avatar, accordion, icon arrays, visualizations, values clarification feature
3	Comprehensive usability testing	3 users+6 experts	Think-aloud protocols Functional prototype testing	Multidevice optimization Refined navigation Specified 4 distinct vaccination decisions
4	Scientific and bilingual review	9 experts	Expert review User journey analysis	Updated content Included French translation Improved interface and instructional text

## Results

### Participant Characteristics

Across the cycles, 45 participants responded to our user testing recruitment efforts, of which 34 completed one iterative user testing phase (cycle 1: n=9, cycle 2: n=22, and cycle 3: n=3). Prospective participants who did not complete the testing did so because they did not wish to finish the study (n=2), were unavailable for any of the times scheduled for testing (n=4), responded after we had already reached saturation in findings (n=2), or for other reasons (n=3). Among the 34 participants, the mean age was 49 (SD 16) years. The majority of our study participants were women, Canadian-born, and English speakers. In total, 11/34, 32% of participants reported an ethnocultural identity other than White. Detailed demographics are shown in Table 2.

**Table 2.** User testing participant demographics across testing cycles (N=34).

Category	Cycle 1 (n=9)	Cycle 2 (n=22)	Cycle 3 (n=3)
Age (years), mean (SD)	47 (16)	50 (16)	52 (21)
Birthplace, n (%)			
Canada	7 (78)	21 (95)	3 (100)
Other	2 (22)	1 (5)	0 (0)
Preferred languages, n (%) (multiple answers allowed)			
French	6 (67)	5 (23)	0 (0)
English	3 (33)	18 (82)	3 (100)
Other	0 (0)	0 (0)	0 (0)
Ethnocultural groups, n (%) (multiple answers allowed)			
Asian—Central (eg, Kazakhstani and Uzbekistani)	0 (0)	0 (0)	0 (0)
Asian—East (eg, Chinese, Japanese, and Korean)	0 (0)	3 (14)	0 (0)
Asian—South (eg, Indian, Pakistani, and Sri Lankan)	0 (0)	1 (5)	0 (0)
Asian—South-East (eg, Malaysian, Filipino, and Vietnamese)	0 (0)	0 (0)	1 (33)
Black—African (eg, Ghanaian, Kenyan, and Somali)	2 (22)	1 (5)	0 (0)
Black—Caribbean (eg, Barbadian and Jamaican)	0 (0)	2 (9)	0 (0)
Black—North American (eg, Canadian and American)	0 (0)	0 (0)	0 (0)
First Nations	0 (0)	0 (0)	0 (0)
Indigenous or aboriginal person from outside of Canada (eg, Native American, Maori, and Quechua)	0 (0)	0 (0)	0 (0)
Inuit	0 (0)	0 (0)	0 (0)
Latin American (eg, Chilean, Mexican, and Salvadorian)	0 (0)	0 (0)	0 (0)
Metis (Metis Nation in Canada)	0 (0)	1 (5)	0 (0)
Middle Eastern (eg, Egyptian, Iranian, and Lebanese)	0 (0)	0 (0)	0 (0)
North African (eg, Moroccan and Tunisian)	0 (0)	0 (0)	0 (0)
White or European (eg, English, Italian, Portuguese, and Russian)	1 (11)	6 (27)	1 (33)
White or North American (eg, Canadian and American)	6 (67)	10 (45)	1 (33)
Other	0 (0)	0 (0)	0 (0)
Prefers not to answer	0 (0)	0 (0)	0 (0)
Disability status, n (%)			
No disability	7 (78)	15 (68)	3 (100)
At least 1 disability	2 (22)	6 (27)	0 (0)
Prefers not to answer	0 (0)	1 (5)	0 (0)
Gender identity, n (%)			
Woman	7 (78)	12 (55)	3 (100)
Man	2 (22)	10 (45)	0 (0)
Indigenous or other cultural gender minority identity (eg, two-spirit)	0 (0)	0 (0)	0 (0)
Something else (eg, nonbinary and gender-fluid)	0 (0)	0 (0)	0 (0)
Prefers not to answer	0 (0)	0 (0)	0 (0)
Highest education level completed, n (%)			
Some elementary school (completed or not)	0 (0)	0 (0)	0 (0)
High school diploma	1 (11)	2 (9)	0 (0)
Apprenticeship or trade certificate or diploma	0 (0)	0 (0)	0 (0)
College or polytechnical school certificate or diploma	1 (11)	7 (32)	0 (0)
University degree, bachelor level or below	3 (33)	10 (45)	2 (67)
University graduate degree (master or doctorate level)	4 (44)	2 (9)	1 (33)
Prefers not to answer	0 (0)	1 (5)	0 (0)
Do not know	0 (0)	0 (0)	0 (0)
Sexual orientation, n (%)			
Straight or heterosexual	8 (89)	17 (77)	3 (100)
Lesbian, gay, or homosexual	1 (11)	1 (5)	0 (0)
Other (options included bisexual, something else, and prefers not to answer)	0 (0)	4 (18)	0 (0)
Health status, n (%)			
Poor	1 (11)	2 (9)	0 (0)
Fair	0 (0)	3 (14)	0 (0)
Good	2 (22)	8 (36)	0 (0)
Very good	5 (56)	7 (32)	2 (67)
Excellent	1 (11)	2 (9)	1 (33)
Public-facing occupation, n (%)			
Yes	2 (22)	5 (23)	0 (0)
No	7 (78)	16 (73)	3 (100)
Prefers not to answer	0 (0)	1 (5)	0 (0)
Provinces and territories, n (%)			
Alberta	1 (11)	0 (0)	0 (0)
British Columbia	0 (0)	1 (5)	0 (0)
Manitoba	0 (0)	4 (18)	0 (0)
New Brunswick	0 (0)	1 (5)	0 (0)
Newfoundland and Labrador	0 (0)	0 (0)	0 (0)
Nova Scotia	0 (0)	0 (0)	0 (0)
Ontario	2 (22)	11 (50)	3 (100)
Prince Edward Island	0 (0)	0 (0)	0 (0)
Quebec	6 (67)	5 (23)	0 (0)
Saskatchewan	0 (0)	0 (0)	0 (0)
Northwest Territories	0 (0)	0 (0)	0 (0)
Nunavut	0 (0)	0 (0)	0 (0)
Yukon	0 (0)	0 (0)	0 (0)
Residing in a rural community, n (%)			
No	—^a^	17 (77)	2 (67)
Yes	—	2 (9)	1 (33)
Do not know	—	3 (14)	0 (0)

a Not available.

### Cycle 1

#### Main Findings

##### Icon-Array Interpretation

All participants (n=9) correctly identified the icon arrays as representing a “group of people” and understood that the colored icons indicated numerical or probabilistic information. However, some participants struggled initially with the precise probabilistic meaning of each icon. Regarding layout preferences, 5 participants preferred the arrays using circular backgrounds, finding them “easier to count” due to clearer spacing and structure. In total, 3 participants favored the shirt-colored arrays because they appreciated seeing “more character detail,” and 1 participant found both layouts equally clear but mentioned that the shirt-colored icons seemed to visually represent more data because of their elongated shape.

##### Provider-Sent Message Framing

When participants reacted to the hypothetical vaccine recommendation message from their primary care provider (Multimedia Appendix 1), 5 of 9 participants expressed neutral feelings toward the message. In total, 2 of 9 participants indicated that receiving such a message would encourage them to consult their provider with further questions. One participant (1/9) found the message reassuring, whereas another (1/9), who had expressed prior vaccine concerns, felt “pressured and uncomfortable.”

##### Vaccine-Confidence Social Media Text

All participants (9/9) appreciated the explanation provided about the rapid vaccine development process (Multimedia Appendix 2). Nevertheless, several participants raised concerns regarding the text’s accessibility and readability. Specifically, 5 of 9 participants thought the text was appropriate mainly for highly educated people or those who already trust the authorities. In total, 3 of 9 participants indicated that the text was too long. Conversely, 3 of 9 participants found this message reassuring. Another 3 of 9 participants (1 English-speaking and 2 French-speaking) reported confusion with the opening sentence, unsure how it related to the subsequent content. Additionally, 1 participant expressed concerns about insufficient transparency regarding potential vaccine side effects.

##### Citizen Panel Recommendations

The citizen panel (n=6) recommended incorporating relatable real-world data into the patient decision aid, automated vaccine selections based on provincial guidelines, and helped to refine visual branding with a logo.

### Changes for the Next Cycle

Following the cycle 1 findings summarized in Table 3, we implemented several targeted modifications to improve the patient decision aid. We explicitly clarified that each icon represented 1 individual in a given population to address confusion around probabilistic visualization. Considering mixed feedback on icon-array layouts, we retained both designs for further testing.

We revised the health care provider message to a more neutral tone, addressing concerns about pressure or discomfort. Finally, to improve the vaccine-confidence content, the structure was reorganized to enhance clarity. We simplified technical language, clarified the opening sentence, and incorporated more transparent information about potential side effects, in line with participants’ expectations regarding tone, accessibility, and completeness.

**Table 3.** Summary of thematic feedback from cycle 1 user testing (n=9) and changes made following citizen panel recommendations.

Theme and key feedback or issue		Participants, n (%)	Resulting modifications
Avatar arrays			
	Interpreted as a “group of people”; needed a clearer explanation that each icon represents 1 individual in a population	9 (100)	Added explanatory text indicating probabilistic meaning
Icon array design preferences			
	Circular array for better spacing	5 (56)	Refined visual layout, used more spacing
	Shirt-colored array for character detail	3 (33)	None (optional alternate design retained for future testing)
Vaccine question framing			
	Neutral feelings about the question	5 (56)	Adjusted question to more neutral wording
	Would consult a health care provider	2 (22)	None; consistent with decision aid concept
	Increased reassurance	1 (11)	None
	Felt pressured, uncomfortable	1 (11)	Wording revised for less prescriptive tone
Vaccine explanation text			
	Effective explanation of rapid vaccine development	9 (100)	Retained main points; reorganized structure
	Perceived as too technical or lengthy	5 (56)	Simplified text and terminology
	Reassuring	3 (33)	Maintained neutral tone
	Confusion with the first sentence	3 (33)	Clarified opening sentence
	Insufficient details on side effects	1 (11)	Emphasized side-effect transparency in revised draft

### Cycle 2

#### Main Findings

##### Overview

During this second round of user testing, participants (n=22) reviewed 2 content variants designed to explain the rapid development of COVID-19 vaccines. The objective was to assess the clarity, effectiveness, and comprehensiveness of each version. Version A contained only illustrations, while version B included photographs and researchers’ names (Multimedia Appendix 4). We randomly assigned 11 participants to version A and 11 to version B. Participants answered questions on message interpretation and identified important unanswered questions about COVID-19 vaccination missing from the explanations (Multimedia Appendix 3).

##### Reactions to the VaxDA-C19 Team Presentation

Participants expressed polarized opinions about version B. In total, 2 participants appreciated the inclusion of researchers’ names and photos, interpreting it as a sign of credibility and transparency. One participant remarked that it was “good to see a Manitoba doctor” involved, while another said that the real faces “gave some credibility.” Others expressed suspicion and discomfort, perceiving the design as overly promotional or politically biased. Participants describe the content using terms like “political PR,” “spin,” or sarcastic phrases such as “a miracle that will solve everything.” Several participants misinterpreted the visuals, believing these scientists developed the vaccines. Some participants perceived the section as an attempt to distance pharmaceutical companies from the vaccine’s origins and described this as reassuring or misleading.

##### Interpretation of Vaccine Explanation Text

In response to the question, “Can you tell me what this text was about?,” most participants described an explanation of how vaccines were developed (version A: 7/11 and version B: 9/11). In total, 5 participants in each group thought that the section highlighted the scientists behind the vaccine development. A small number of participants said that the message aimed to counter vaccine skepticism or clarify funding sources. Table S1 in Multimedia Appendix 6 summarizes how participants interpreted the vaccine explanation text.

##### Reactions to the Vaccine Explanation Text

During this round of testing, participants (n=22) provided varied emotional reactions and interpretations regarding the explanatory text on rapid COVID-19 vaccine development. In total, 2 participants perceived a clear division between the scientists or authorities and the public, illustrated by expressions of suspicion or detachment such as “The people who created it are vouching for it,” and referring to authorities as “Them.”

Some participants identified important information gaps, particularly the lack of details on vaccine effectiveness, side effects, and characteristics of clinical trial participants. For instance, a participant remarked that the vaccine was presented as “a miracle that will solve everything,” while another expressed nuanced skepticism: “I know exactly how it’s done, and I take some and leave some [...] I’m a bit perplexed.” In total, 2 participants specifically indicated confusion or misunderstanding about the sentence stating, “Other vaccines are still being tested in places with fewer COVID-19 cases.” One described needing more time to understand the phrase clearly, while the other explicitly found it ambiguous.

One participant strongly criticized the text as too political and not scientific, disputing claims about vaccine availability and the accuracy of funding statements. Another felt that the text insufficiently emphasized the societal consequences of refusing vaccination, remarking strongly that, “It’s basically a crime if people are not taking this seriously.”

Finally, one participant noted perceived contradictions within the explanation, particularly regarding how previous coronavirus research aligns with the rapid vaccine development and funding during the pandemic. This participant also highlighted inconsistencies in public health messages from different provincial health authorities. Table S2 in Multimedia Appendix 6 summarizes the main emotional reactions and comprehension issues identified.

##### Unanswered Questions

Table S3 in Multimedia Appendix 6 summarizes the types of questions participants felt remained unanswered after reading the vaccine explanation text. These focus on vaccine mechanisms, COVID-19–specific information, distribution and eligibility, safety, and research.

### Changes for the Next Cycle

Building on participant feedback in cycle 2, we made several targeted changes to the next iteration of the VaxDA-C19 decision aid to enhance its clarity, transparency, and trustworthiness. The photos and names of scientists were moved to a dedicated “About Us” section to reduce perceptions of promotional content and clarified their roles to reduce confusion. While keeping the explanatory text detailed, we simplified complex terms to increase accessibility. In response to recurring concerns and unanswered questions about vaccine technology, safety, variants, and side effects, we also expanded the FAQs section.

### Cycle 3

#### Main Findings

During cycle 3, we conducted a third round of user testing (n=3) and expert consultation (n=6) to assess navigation, usability, and comprehension across devices. Table S4 in Multimedia Appendix 6 presents a thematic summary of participant feedback.

##### Navigation and Usability Improvements

Participants (users: n=3 and experts: n=6) identified significant navigation issues, notably difficulties finding the “Next” button, which impaired smooth transitions between sections. They also recommended clearer labeling and explicit instructions to clarify interactions with pages involving comparative vaccine selection and slider functionalities.

##### Information Transparency and Accessibility

Participants emphasized the need for simplified medical terminology and a more transparent presentation of vaccine efficacy and side effects data. They noted that clear, immediate comparative data would be crucial, particularly important for parents evaluating COVID-19 vaccination for children.

##### Interactive Features Optimization

Interactive elements, such as avatar creation and values clarification sliders, received mixed responses. Participants initially perceived avatar creation positively, but 1 participant found creating multiple avatars to form a community as unnecessary or excessively time-consuming. One participant had a preference for factual information rather than interactive elements or animations. Sliders were occasionally confusing due to unclear functionality interactions, dull colors, and poorly defined medical terms.

##### Insights From External Experts

Five health care professionals and medical decision-making researchers provided feedback at a medical decision-making conference. This feedback included recommendations for improved mobile navigation, structured vaccine selection menus, refined content spacing, and modal placements. A subsequent external reviewer (n=1) with expertise in UX recommended adding a step-by-step user guide and informative pop-ups to support user interactions.

### Changes for the Next Cycle

Following cycle 3, we implemented targeted modifications to improve navigation, content clarity, and overall UX in VaxDA-C19. We enhanced button visibility and repositioned key navigation elements, adding clear user instructions to support a smoother transition. To increase content accessibility and transparency, we simplified medical terminology and added comparative data on vaccine efficacy and side effects directly within relevant sections. To streamline interactivity, we introduced an avatar bypass option and clarified the functionality of values clarification sliders through revised instructions and enhanced visual design. External expert recommendations were also applied to improve mobile navigation, refine spacing, structure vaccine menus more clearly, and integrate contextual guidance through informative pop-ups.

### Cycle 4

#### Main Findings

Cycle 4 consisted of a comprehensive expert review (n=9), focusing on usability, clarity, bilingual implementation, and overall application refinements as shown in Table S5 in Multimedia Appendix 6.

##### High-Priority Usability Improvements

Experts (n=9) identified several issues affecting clarity and UX. These included irrelevant vaccine comparisons for children younger than 12 years of age, unclear user instructions, confusing avatar placement, navigation difficulties on the mobile interface, and incomplete French implementation. Experts also suggested clarifying the decision-making context consistently across the web-based application (eg, clearly indicating options such as “I don’t know”) and recommended optimizations for animations and interactive sliders to reduce confusion and enhance usability.

##### Moderate-to-Low Priority Feedback

Additional recommendations included improving free navigation across sections, clearly displaying institutional logos and contact information, enhancing visual spacing for provincial selection buttons, and refining icon-array color schemes. Experts also proposed custom speed and pause features for animations, reducing overall text density, and simplifying vaccine comparison visuals. Due to technical and feasibility constraints, we recorded moderate-to-low priority feedback for future updates. Textbox 3 summarizes the key points from the design and development process.

Textbox 3. **Key points from usability testing results.**

Navigation and interface usability: Participants consistently highlighted the need for clear, intuitive, and easily navigable interfaces. Regular usability checks using small iterative samples allowed the research team to quickly identify critical navigation barriers. Participants recommended explicit labeling of navigation buttons (such as the “Next” button), logical repositioning of interactive elements, and improved visibility of control. These adjustments may improve user satisfaction and reduce cognitive load.

Information transparency and accessibility: Participants frequently emphasized the importance of clear, concise, and transparent information on vaccine efficacy and side effects. The research team anticipated that simplifying medical language and providing immediate comparative information could enhance user engagement, comprehension, and trust. Participants identified addressing transparency around sensitive health decisions, such as pediatric vaccination, as important to increase the usability and credibility of the patient decision aid.

Interactive features optimization: Participants suggested explicitly defining medical terms, clarifying instructions for using interactive elements (avatars and values clarification sliders), optimizing slider functionalities, and offering optional bypass features for less nonessential interactive steps. The research team incorporated these recommendations to reduce perceived cognitive burden and potentially enhance user experience, though further validation remains necessary.

Responsive content adjustments: User feedback identified specific gaps in information (eg, vaccine efficacy, technology, clinical trial, and distribution logistics) and highlighted areas susceptible to misunderstanding or skepticism (eg, funding sources and researchers’ roles). Participants recommended expanding and refining frequently asked questions, clearly explaining scientific roles, and transparently communicating funding and clinical processes. The research team implemented these content adjustments to address user skepticism, improve comprehensiveness, and enhance perceived relevance.

Multidisciplinary and expert reviews: Feedback from multidisciplinary perspectives, including health care professionals, user experience specialists, and citizen panels, helped refine the web-based patient decision aid’s usability and scientific content. The research team ranked experts’ recommendations according to their overall importance and implemented all high and medium priorities, such as improved mobile navigation, a restructured vaccine selection process, and clearer scientific explanations. These refinements aimed to enhance clarity, accessibility, and user acceptance, although further formal validation of their impact is required.

### Implementation of the Final Version of VaxDA-C19

**Figure 4.**
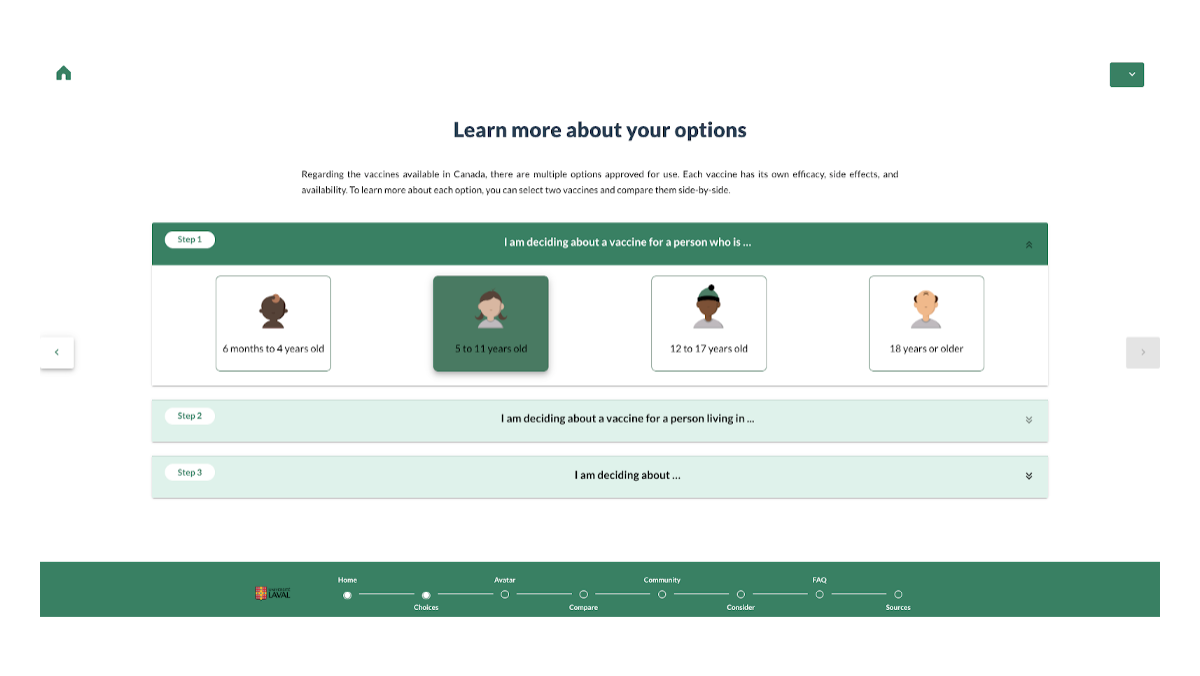
Visual representation of the VaxDA-C19’s choices page.

VaxDA-C19 evolved from an initial Google Slides mock-up into a fully functional, bilingual web-based application (Figures4  5). Key technical improvements include transitioning to a responsive HTML5 and JavaScript architecture, with real-time content synchronization (Multimedia Appendix 5). Major features include automated visualizations for herd immunity, refining comparative interactive features, and enhanced icon arrays. These refinements ensure that VaxDA-C19 remains human-centered, responsive to evolving vaccination guidelines, while improving usability and user trust.

**Figure 5.**
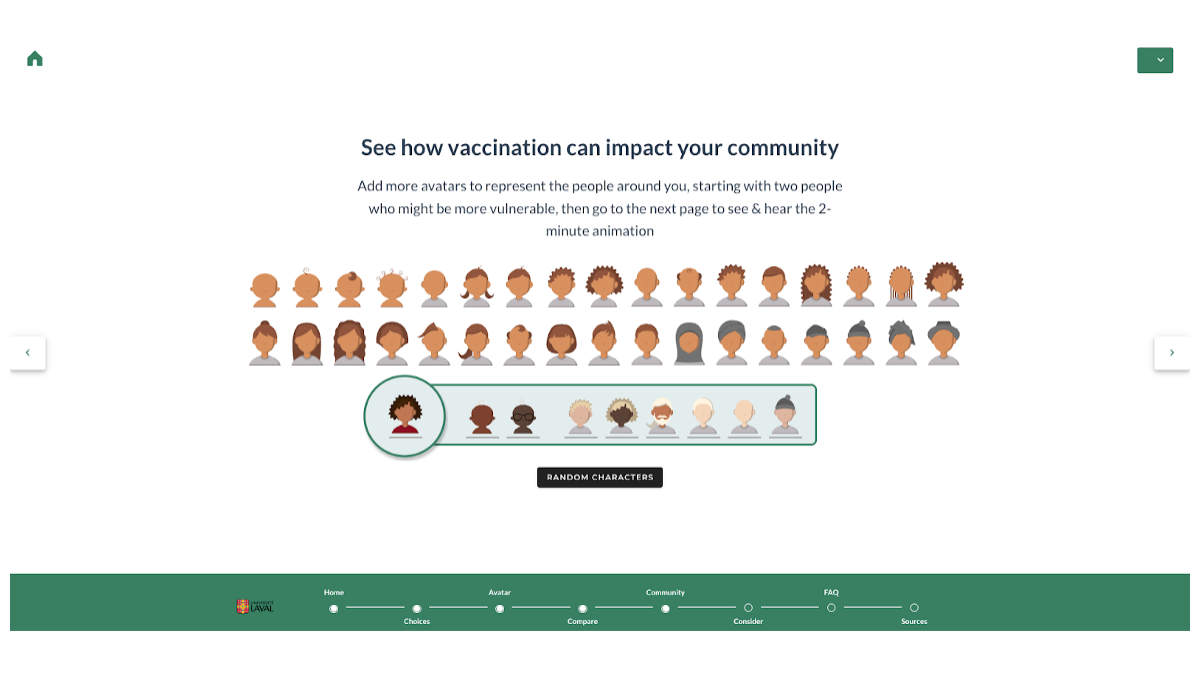
Selected views from the final version of VaxDA-C19.

## Discussion

### Principal Findings

This study aimed to iteratively develop and refine a bilingual (English and French) web-based patient decision aid (VaxDA-C19) to help people in Canada make evidence-informed, values-congruent decisions about COVID-19 vaccination. VaxDA-C19 includes interactive components such as customizable avatars, personalized icon arrays constructed out of those avatars, an optional, animated, theory-based visualization of community immunity, and a values clarification method. We included these features based on their capacity to facilitate effective communication, optimize cognitive load, enhance empathy, and provide clear, evidence-based information on vaccine options, benefits, and risks [32,48,49,52,61-63]. We present 3 principal findings from this work.

First, a recurrent theme in participant feedback was the need for more detailed and transparent information about vaccine efficacy, variant coverage, immunity duration, and the inclusivity of clinical trials. To address these concerns, enhance transparency, and hopefully reinforce trust in the information presented, rather than offering a brief list of references in a separate section at the end of the document or website, as is commonly done in patient decision aids, we integrated explicit links to peer-reviewed studies and health authority documents immediately following each statement. We used the word “citation” for each reference and linked directly to open-access resources whenever possible. We also designed visual strategies, such as including images of scientists, to increase credibility [65-67]; however, responses to this approach varied. Some participants found this approach reassuring, while others perceived it as manipulative or politically motivated. To ultimately mitigate these concerns, we removed images and prioritized emotional neutrality and transparent, nonjudgmental communication [1,4,15,68-70]. This approach aligns with the trust determination theory, which emphasizes how credibility, inclusivity, and clarity foster public confidence and reduce skepticism [71]. Similarly, the extended parallel process model highlights how fear, distrust, and perceived governmental or institutional biases influence message reception, potentially diminishing the effectiveness of health communication [72].

Second, rather than seeking an elusive one-size-fits-all information presentation, presenting a simple overview of essential points and allowing people to easily access more detail when they want it may better serve a wider range of information needs. People with lower health literacy, who often express greater vaccine hesitancy, find more transparent explanations of risks and benefits particularly helpful [68,73-75]. Some participants in our study similarly emphasized the importance of clear, simplified explanations, particularly for individuals with varying levels of health literacy and also for people who lack the time or interest to delve deeply into information about vaccines. However, as noted earlier in our first point, other participants wanted far more detail. These tensions between some people’s desires for comprehensive information and others’ desires for more succinct information highlighted the complexity inherent in balancing simplicity and visual appeal with informational accuracy [76,77]. To address these competing priorities, we revised medical terminology, restructured content for better readability, and ultimately presented a summary of key evidence to all users, followed by accordion menus, which allow users to expand sections to read more about a given topic. By giving people control over the amount of detail they receive, we hope VaxDA-C19 will serve the needs of people who want a summary of essential points as well as people whose questions are not answered by simplified public health messages. Future research should continue to examine optimizing content presentation to provide sufficient detail without overwhelming users [78].

Third and finally, specific challenges associated with developing a patient decision aid for COVID-19 vaccination included a novel pathogen that launched a global pandemic, novel vaccine technology, continuously evolving evidence about vaccine effectiveness and adverse events following immunization, changing vaccine products and authorizations, and evolving recommendations for use. The fast-evolving COVID-19 vaccination landscape required proactive content management. We used flexible structures like JSON templates to enable timely updates aligned with new evidence and public health recommendations. This technical adaptability, combined with sufficient time commitments on the part of team members, should help us ensure that the VaxDA-C19 will remain scientifically accurate, while also facilitating version control, scalability, and potential future reuse for patient decision aids about other vaccine-preventable diseases.

### Comparisons With Existing Literature

The COVID-19 pandemic led to the rapid development of digital patient decision aids aimed at addressing vaccine hesitancy, which intensified due to misinformation and information overload (infodemic) [79]. Several countries, including France [80], the Netherlands [81], and Australia [82], introduced digital tools to support informed decision-making. Compared to static informational approaches, these interactive decision aids improved user knowledge, reduced decisional conflict, and increased satisfaction with the decision-making process [79,81]. Despite these benefits, major challenges persist, including potential biases in content presentation, variations in complexity, and inconsistent accessibility, all of which affect usability and effectiveness [79,81].

International evaluations identified key factors that contribute to the success of digital patient decision aids. Co-design approaches that involve health professionals and target populations enhance relevance and usability. Regular updates ensure content accuracy and alignment with evolving public health guidelines. User-friendly interfaces and personalization based on individual risk assessments further support engagement and informed decision-making [79-82]. Certain digital patient decision aids have also integrated practical features, such as direct scheduling links for vaccination appointments, to facilitate action [79,81]. Similarly, in our study, multiple cycles of qualitative usability testing within an iterative, human-centered approach helped identify and address navigation, comprehension, and usability issues early in development. Small-scale usability tests effectively detected key issues, allowing for rapid refinements. Cognitive ergonomics literature supports this strategy, indicating that explanatory texts provide more explicit contextual cues and facilitate information processing, particularly for individuals with lower health literacy [83-85].

Limited linguistic and cultural adaptability restricts the reach of many existing digital patient decision aids, particularly among culturally and linguistically diverse populations [79,81]. VaxDA-C19 aims to address this limitation initially through its bilingual content in English and French, the official languages of Canada. Following evaluation in an online randomized controlled trial, we also plan to translate it into other major languages spoken in Canada.

Although digital patient decision aids may influence vaccine intentions, evidence directly linking these tools to increased vaccine uptake remains incomplete. Ongoing randomized controlled trials, such as SMART-DA in Korea [86], aim to assess their impact on key outcomes, including vaccine intentions, knowledge retention, decisional conflict, and emotional responses. Findings suggest that patient decision aids improve preventive health behaviors by enhancing decision quality rather than by applying persuasive messaging [87,88].

Our study aligned with previous research, suggesting that effective communication in patient decision aids such as VaxDA-C19 requires careful consideration of emotions, trust, and perceived biases [1,4,10,89]. Emotional responses play an important role in how individuals interpret vaccine-related messages [1,15]. In our study, some participants expressed skepticism, anxiety, or distrust, particularly if they perceived wording as being politically charged, overly promotional, ambiguous, or institutionally biased. This finding aligns with other studies [90], and these reactions overall align with Sandman’s risk=hazard + outrage model, which posits that public outrage or mistrust can overshadow objective risk assessments, leading to defensive responses such as rejection or disengagement [4,18,90,91]. Preexisting attitudes toward vaccines may also influence people’s reactions to interventions such as VaxDA-C19. Future research should incorporate such considerations into study design.

Our study also confirmed that intuitive navigation, visual clarity, and balanced content presentation played essential roles in usability. These findings align with existing literature, which demonstrates that interactive and personalized elements improve engagement and comprehension in digital health interventions [52,92,93].

### Strengths and Limitations

Our study has 2 main limitations. First, while recruiting user testing participants through tailored Facebook advertisements allowed us to reach people living in different parts of Canada with diverse backgrounds and who are online and therefore potential users of a web-based patient decision aid, no recruitment platform can perfectly represent the broader population. We note that our user testing participants were, as a group, slightly older than the broader group of Facebook users in Canada. Specifically, among Facebook users in Canada, the largest age group (around 25%) is between 25 and 34 years, with the next largest group (19%) being 35 to 44 years [94]. We may have recruited more people older than these groups due to increasing interest in COVID-19 vaccines with age. We note that the age group we recruited was approximately representative of the desired population for a patient decision aid about COVID-19 vaccines. Older people in Canada were already receiving strong recommendations to obtain the vaccine, whereas people in the age range of our user testing participants were likely to be both candidates themselves and may also be parents of children who were candidates for COVID-19 vaccination at the time of the study. Our study participants also included more people with higher levels of education than the general population in Canada. People with more education may seek more information about health compared to people with less education. Additionally, we note that, as a group, participants included a substantial proportion of people without a college or university degree. In other words, although there were many people with higher levels of education in our study, there were also many people without such education. No one intervention can be everything to everyone; our goal with this intervention was to provide a middle ground between the simplified information available on public health websites and the detailed information buried in technical documents and academic literature. Because vaccination campaigns are most successful when they reach as many people as possible, an approach with different components for different subpopulations (eg, an online patient decision aid for people with enough motivation to seek more information, community-based education meetings for people who trust community leaders, and simple information for people who have no questions and simply want to know where to get their vaccine) is likely to achieve the best population-level protection. Second, the challenge of balancing comprehensive content with cognitive load remains potentially unresolved, particularly in adapting information for users with varying levels of health literacy. While brief, simple messaging can be most effective in some contexts and for some people, the resulting lack of detail and missing explanations can be a problem for others. Variability in patient decision aid formats, development methodologies, and user preferences suggests that no single approach fits all contexts [25,95]. We therefore chose to prioritize user control over information presentation, trusting that many people will know what they need. Nevertheless, additional research should investigate these aspects further to refine content presentation and improve the effectiveness of digital patient decision aids.

Our study also has 2 main strengths. First, we used a rigorous iterative design based on human-centered methodologies, a multidisciplinary approach, bilingual validation, and structured engagement of a range of interested parties, including user testing, a citizen panel, UX interviews, and expert reviews. The collaboration between a diversity of researchers, UX specialists, members of the public, and health care professionals helped refine the patient decision aid, contributing to its scientific reliability and usability. User testing participants from different backgrounds offered different responses and reactions, providing a range of feedback to which we were able to respond. Complementing the contributions of these members of the public who participated in user testing and saw VaxDA-C19 at a single time point, members of the citizen panel saw VaxDA-C19’s evolution over time and were able to comment at a higher level on how well or poorly the design was responding to its original goals as well as the evolving pandemic context. A wide range of experts brought insights from related projects, literature, and guidelines and also lent their expertise to verify VaxDA-C19’s content and design. Systematically integrating all these people’s feedback on an ongoing basis allowed for continuous adjustments. Second, we believe that VaxDA-C19 fills an unmet need by bridging the gap between highly technical vaccine information and highly simplified public health messaging. As COVID-19 vaccines continue to be offered to people in Canada, we hope to help support high-quality decision-making, thus contributing to the health of people in Canada and to public health more broadly. The platform itself may also be adapted to other vaccines.

We plan to assess the effects of VaxDA-C19 in an online randomized controlled trial (ISRCTN91743566). Specifically, participants who are eligible to receive COVID-19 vaccines will be randomly assigned to 1 of 3 arms: a control group receiving no additional information, a usual information group directed to the Public Health Agency of Canada’s COVID-19 vaccination web pages, or an intervention group receiving access to VaxDA-C19. We will assess effects on the primary outcome of vaccination intentions and secondary outcomes: knowledge, emotions, decisional conflict, vaccine uptake, and decisional regret. We will also examine effects on trust in the information provided, comparing only between the usual information and VaxDA-C19. Finally, we will conduct a follow-up study 3‐6 months later to ascertain whether or not participants received COVID-19 vaccines.

### Conclusions

VaxDA-C19 is an online patient decision aid designed and developed to help people in Canada make evidence-informed, values-congruent decisions about COVID-19 vaccines on an ongoing basis. An iterative, human-centered design approach and a multidisciplinary team bringing diverse perspectives led to a flexible application that we hope will prove useful for years to come.

## Supplementary material

10.2196/86283 Multimedia Appendix 1 User testing interview guide—cycle 1.

10.2196/86283 Multimedia Appendix 2 Proposed social media text on vaccine confidence—cycle 1.

10.2196/86283 Multimedia Appendix 3 User testing interview guide—cycle 2.

10.2196/86283 Multimedia Appendix 4 Vaccine explanation text storyboard used in cycle 2.

10.2196/86283 Multimedia Appendix 5 Technical development and version control workflow for VaxDA-C19.

10.2196/86283 Multimedia Appendix 6 Qualitative findings from user testing and expert review.

10.2196/86283 Checklist 1 DEVELOPTOOLS reporting checklist.

10.2196/86283 Checklist 2 SRQR reporting checklist for qualitative studies.

## Abbreviations

FAQ: frequently asked question
SRQR: Standards for Reporting Qualitative Research
UX: user experience
