# Supplementary material for: Interactive, Personalized Patient Decision Aid for COVID-19 Vaccination in Canada: User-Centered Design Approach
Source: JMIR Hum Factors. 2026 Apr 16;13:e86283. doi: 10.2196/86283 (PMC13086185; doi:10.2196/86283)
Supplement: Multimedia Appendix 1 [file humanfactors-v13-e86283-s001.pdf]

User testing interview guide - cycle 1

**English script**	**French script**	
Thank you very much for taking the time to meet me. This is in the first phase of our project and we hope to meet with about 20 people who will be able to give us their opinion on communication materials related to COVID-19.	Merci beaucoup de prendre le temps de me rencontrer. Ce projet en est à sa première phase et nous espérons rencontrer une vingtaine de personnes qui pourront nous donner leur avis sur des outils de communication par rapport à la COVID-19.	
I will first explain in more detail what the project is all about, and then you can confirm whether or not you want to participate.	Je vais d'abord vous expliquer plus en détail en quoi consiste le projet, vous pourrez ensuite me confirmer si vous voulez participer ou non	
[Share your screen by making sure to click "share audio”]	[Partager votre écran en vous assurant de cliquer “partager l'audio”]	
[Copy the link to the consent form in the presentation and send it via chat to the participant.]<br><br>[Read consent form (outline)]<br><br>[Let the person complete the surveys]	[Copier le lien vers le formulaire de consentement dans la présentation et l'envoyer par chat au participant.]<br><br>[Lire formulaire de consentement (grandes lignes)]<br><br>[Laisser la personne compléter les sondages]	
Thank you. I'm going to start recording the session, then I'm going to show you prototypes tools that I would like to have your opinion on.	Merci. Je vais démarrer l'enregistrement de la séance, puis je vais vous montrer des prototypes d'outils sur lesquels j'aimerais avoir votre avis.	
Here are two pairs of similar images. What do you think they mean?<br>Is there one pair that seems easier to read than the other?	Voici deux paires d'images semblables. Comment interprétez-vous ces images ?<br>Y a-t-il une paire qui vous semble plus facile à distinguer ?	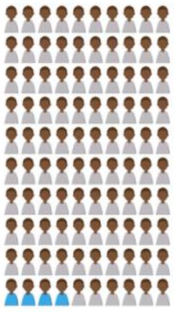 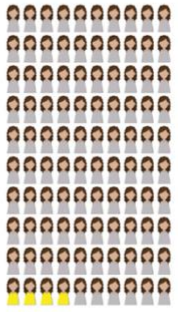

		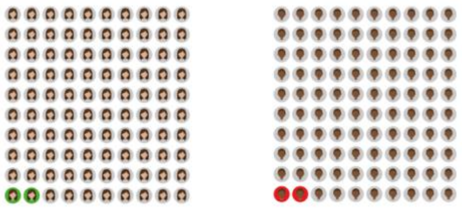
Does your primary care provider have a way to send you messages either via email or a patient portal?<br><br>[if yes:]<br><br>Imagine your primary care health professional (e.g., family doctor, nurse practitioner) sends you a message like, “I got my COVID-19 vaccine when I was eligible. Now that you are eligible, I'd like to support you by answering any questions you might have. Here's a link with some information: link. Please send me a message if you have more questions.” How would that make you feel? How might you react or respond?<br><br>[if not:]<br><br>Imagine you are seeing your primary care health professional (e.g., family doctor, nurse practitioner) and they tell you, “I got my COVID-19 vaccine when I was eligible. Now that you are eligible, I'd like to support you by answering any questions you might have.” How would that make you feel? How might you react or respond?	Est-ce que votre fournisseur principal de soins de santé (votre médecin ou votre infirmière praticienne) vous envoie des messages, soit par courriel ou un autre type de messagerie en ligne ?<br><br>[si oui :]<br>Imaginez que votre professionnel de la santé (par exemple, votre médecin de famille, votre infirmière praticienne) vous envoie un message du genre : "J'ai reçu mon vaccin COVID-19 quand j'étais éligible. Maintenant que vous êtes éligible, j'aimerais vous soutenir en répondant à toutes vos questions. Voici un lien avec quelques informations : lien. Veuillez m'envoyer un message si vous avez d'autres questions".<br><br>Qu'en penseriez-vous ? Comment pourriez-vous réagir ou répondre ?<br><br>[si non :]<br><br>Imaginez que vous voyiez votre professionnel de santé (par exemple, un médecin de famille, une infirmière praticienne) et qu'il vous dise : "J'ai reçu mon vaccin COVID-19 quand j'étais éligible. Maintenant que vous êtes éligible, j'aimerais vous soutenir en répondant à toutes vos questions". Qu'en penseriez-vous ? Comment réagiriez-vous ?	Appendix 3: Storyboard for Communicating Vaccine Confidence in the COVID-19 Vaccine Decision Aid
I'm sure you heard that vaccines are now available for COVID-19. I will show you a text about this that we'd	J'imagine que vous savez que des vaccins pour la COVID-19 sont maintenant disponibles. Je vais vous montrer un texte à ce propos	

want to disseminate through social media.<br><br>Could you give me your opinion about this text?<br><br>If you saw this type of text on social media, would you read it? Why or Why not?	qui pourrait être diffusé sur les médias sociaux.<br><br>J'aimerais que vous me donniez votre avis sur ce texte.<br><br>Si vous voyiez ce type de texte sur les médias sociaux, le liriez-vous ? Pourquoi ?	
That concludes our study, thank you very much for participating. We will send you 40$ by Interac e-Transfer. Do you know how it works?<br><br>What email address or cell phone number should I send it to?<br><br>[Confirmed]<br><br>The answer to the security question will be **COVID-19** (uppercase and without hyphens).<br>Goodbye!	Cela met fin à notre étude, je vous remercie beaucoup d'avoir participé. Nous allons vous envoyer 40 $ par transfert Interac. Savez-vous comment cela fonctionne ?<br><br>À quelle adresse email ou numéro de cellulaire devrais-je vous le faire parvenir ?<br><br>[Confirmer]<br><br>La réponse à la question de sécurité sera **COVID-19** (en majuscule et sans trait d'union).<br>Au revoir!	
