# Supplementary material for: Interactive, Personalized Patient Decision Aid for COVID-19 Vaccination in Canada: User-Centered Design Approach
Source: JMIR Hum Factors. 2026 Apr 16;13:e86283. doi: 10.2196/86283 (PMC13086185; doi:10.2196/86283)
Supplement: Multimedia Appendix 2 [file humanfactors-v13-e86283-s002.pdf]

Proposed social media text on vaccine confidence - cycle 1

Text and image description for an intervention that will be produced as both a series of images (for use on platforms where multiple images work best; e.g., tweet thread, Instagram carousel) or an infographic (e.g., Facebook, Pinterest)

**English text**
9 women can't make a baby in 1 month, no matter how well they work together!
But when a bunch of the world's best scientists work together, with enough research funding, motivated by a pandemic that is disrupting lives around the world, vaccine development can happen faster than usual.
Making a new vaccine usually involves months or years of waiting time. Waiting to get more research funding. Waiting for enough people to sign up for studies that make sure the vaccines are safe and work well. Waiting for approvals from independent regulatory authorities before starting to produce the vaccine for distribution.
Usually, this waiting happens because the need for the vaccine isn't considered urgent enough for everyone to drop everything else and make the new vaccine everyone's top priority. But COVID-19 has been a different story. It has been a priority around the world.
To make COVID-19 vaccines, they cut out a lot of the waiting. But they didn't cut corners. All the safety checks are still being done. When COVID-19 vaccines are approved by Health Canada (Canada's independent agency, staffed by top-notch scientists and not affiliated to any political party) you can trust that they are safe and they work.
We are Canadian scientists, doctors, nurses, and experts in health and vaccines. None of us work for pharmaceutical companies. We don't accept pharmaceutical funds either. We will get the COVID-19 vaccines for ourselves as soon as they are approved by Health Canada and available to us. Once they have been tested and approved for children, our children will get the vaccines, too.
Still have questions about COVID-19 vaccines and don't know where to ask? We want to use our scientific training to help answer your questions. Send us your question here. We will do our very best to answer as many as we can.
**French text**
9 femmes ne peuvent pas faire un bébé en 1 mois, même si elles travaillent bien ensemble !
Mais lorsque les meilleurs scientifiques du monde travaillent ensemble, avec suffisamment de fonds pour la recherche et motivés par une pandémie qui perturbe des vies dans le monde entier, le développement de vaccins peut se faire plus rapidement que d'habitude.
La fabrication d'un nouveau vaccin implique généralement des mois ou des années d'attente. Attendre pour obtenir davantage de fonds pour la recherche. Attendre qu'un nombre suffisant de personnes se portent volontaires pour participer à des études visant à garantir la sécurité et l'efficacité des vaccins. Attendre l'approbation des autorités avant de commencer à produire le vaccin pour la distribution.
En général, tous ces délais sont causés parce que les besoins ne sont pas considérés comme suffisamment urgents pour que tout le monde fasse du nouveau vaccin sa priorité absolue Mais dans le cas de la COVID-19, c'est une autre histoire. La création d'un vaccin a été une priorité dans le monde entier
Les délais ont été moins longs pour fabriquer les vaccins contre la COVID-19, mais cela ne veut pas dire que des raccourcis ont été pris. Tous les contrôles de sécurité sont faits. Lorsque les vaccins contre la COVID-19 sont approuvés par Santé Canada (l'agence indépendante du Canada, composée de

scientifiques de haut niveau et non affiliée à un parti politique), vous pouvez être convaincus qu'ils sont sûrs et qu'ils fonctionnent.
Nous sommes des scientifiques, des médecins, des infirmières et des experts canadiens en matière de santé et de vaccins. Aucun d'entre nous ne travaille pour des sociétés pharmaceutiques. Nous n'acceptons pas non plus de fonds des compagnies pharmaceutiques. Nous nous ferons vacciner contre la COVID-19 dès que les vaccins seront approuvés par Santé Canada et mis à notre disposition. Une fois qu'ils auront été testés et approuvés pour les enfants, nos enfants recevront, eux aussi, les vaccins.
Vous avez encore des questions sur les vaccins contre la COVID-19 et vous ne savez pas où les poser ? Nous voulons utiliser notre formation scientifique pour vous aider à répondre à vos questions. Envoyez-nous votre question ici. Nous ferons de notre mieux pour répondre à autant de questions que possible.
