# Supplementary material for: Interactive, Personalized Patient Decision Aid for COVID-19 Vaccination in Canada: User-Centered Design Approach
Source: JMIR Hum Factors. 2026 Apr 16;13:e86283. doi: 10.2196/86283 (PMC13086185; doi:10.2196/86283)
Supplement: Multimedia Appendix 3 [file humanfactors-v13-e86283-s003.pdf]

User testing interview guide - Cycle 2

English script	French script
Thank you very much for taking the time to meet me. This is in the first phase of our project and we hope to meet with about 20 people who will be able to give us their opinion on communication materials related to COVID-19.	Merci beaucoup de prendre le temps de me rencontrer. Ce projet en est à sa première phase et nous espérons rencontrer une vingtaine de personnes qui pourront nous donner leur avis sur des outils de communication par rapport à la COVID-19.
I will first explain in more detail what the project is all about, and then you can confirm whether or not you want to participate.	Je vais d'abord vous expliquer plus en détail en quoi consiste le projet, vous pourrez ensuite me confirmer si vous voulez participer ou non
[Share your screen by making sure to click "share audio"]	[Partager votre écran en vous assurant de cliquer "partager l'audio"]
[Copy the link to the consent form in the presentation and send it via chat to the participant.]<br><br>[Read consent form (outline)]<br><br>[Let the person complete the surveys]	[Copier le lien vers le formulaire de consentement dans la présentation et l'envoyer par chat au participant.]<br><br>[Lire formulaire de consentement (grandes lignes)]<br><br>[Laisser la personne compléter les sondages]
Thank you. I'm going to start recording the session, and then I'll show you prototype tools that I would like to get your opinion on.	Merci. Je vais démarrer l'enregistrement de la séance, puis je vais vous montrer des prototypes d'outils sur lesquels j'aimerais avoir votre avis.
In your own words, can you tell me what this text was about?<br><br>Why did we get these vaccines so quickly?<br><br>What important questions about COVID-19 vaccines aren't answered by this?	Pouvez-vous me résumer ce texte dans vos propres mots ?<br><br>Pourquoi les vaccins contre la COVID-19 ont été faits aussi rapidement ?<br><br>Quelles autres questions importantes à propos des vaccins contre la COVID-19 n'ont pas été expliquées dans ce texte ?
That concludes our study, thank you very much for participating. We will send you 40$ by Interac e-Transfer. Do you know how it works?<br><br>What email address or cell phone number should I send it to?<br><br>[Confirmed]<br><br>The answer to the security question will be **COVID-19** (uppercase and without hyphens).<br><br>Goodbye!	Cela met fin à notre étude, je vous remercie beaucoup d'avoir participé. Nous allons vous envoyer 40$ par transfert interac. Savez-vous comment cela fonctionne ?<br><br>À quelle adresse email ou numéro de cellulaire devrais-je vous le faire parvenir ?<br><br>[Confirmer]<br><br>La réponse à la question de sécurité sera **COVID-19** (en majuscule et sans trait d'union)<br><br>Au revoir !
