# Supplementary material for: Interactive, Personalized Patient Decision Aid for COVID-19 Vaccination in Canada: User-Centered Design Approach
Source: JMIR Hum Factors. 2026 Apr 16;13:e86283. doi: 10.2196/86283 (PMC13086185; doi:10.2196/86283)
Supplement: Multimedia Appendix 4 [file humanfactors-v13-e86283-s004.pdf]

Vaccine explanation text storyboard used in cycle 2

**English script**	**French script**	
Making a new vaccine usually takes years. But when a bunch of the world's best scientists are able to work together, motivated by a pandemic that is disrupting lives around the world, vaccine development can happen faster than usual.<br><br>Canada got COVID-19 vaccines faster than usual for 5 reasons.	La fabrication d'un nouveau vaccin prend généralement des années. Mais lorsqu'un groupe des meilleurs scientifiques du monde est capable de travailler ensemble, motivé par une pandémie qui perturbe des vies dans le monde entier, le développement d'un vaccin peut se faire plus rapidement que d'habitude.<br><br>Le Canada a obtenu les vaccins COVID-19 plus rapidement que d'habitude pour cinq raisons.	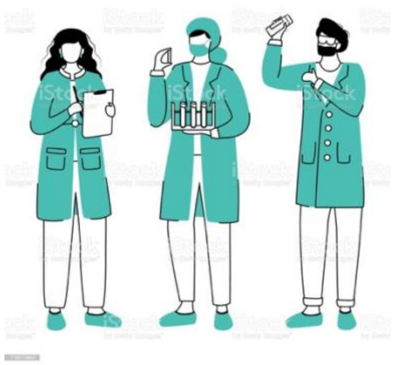
**17 years of research on coronaviruses**<br><br>Ever since the SARS pandemic in 2003 (which affected Toronto and other places in Canada) scientists have been working on understanding coronaviruses and developing vaccines that could prevent the diseases that coronaviruses cause.	**17 ans de recherche sur les coronavirus**<br><br>Depuis la pandémie de SRAS en 2003 (qui a touché Toronto et d'autres endroits au Canada), les scientifiques travaillent à la compréhension des coronavirus et à la mise au point de vaccins qui pourraient prévenir les maladies causées par les coronavirus.	
**Lots of research funding**<br><br>Developing new vaccines is often slower than it could be because scientists have to apply for funding to do the science. There often isn't enough research funding for all the science that could be done, so it often takes lots of tries at submitting grants before finally securing funding. In the case of COVID-19, governments and other sources made substantial research funding available, allowing many scientists to quickly start studies. For example, the government of Canada allocated funds to the Canadian Institutes of Health Research and to Canadian	**Un financement généreux pour la recherche**<br><br>Le développement de nouveaux vaccins est souvent plus lent qu'il ne pourrait l'être, car les scientifiques doivent demander des fonds pour faire leurs recherches. Il n'y a souvent pas assez de fonds de recherche pour tout le travail qui doit être effectué, de sorte qu'il faut souvent faire de nombreux essais pour soumettre des demandes de subvention avant d'obtenir un financement. Dans le cas de la COVID-19, les gouvernements et d'autres organismes ont offert des fonds de recherche substantiels, permettant à de nombreux scientifiques de commencer rapidement leurs études. Par	

vaccine developers like VIDO in Saskatoon and Medicago in Quebec City.	exemple, le gouvernement du Canada a alloué des fonds aux Instituts de recherche en santé du Canada et aux développeurs de vaccins canadiens comme VIDO à Saskatoon et Medicago à Québec.	
**COVID-19 studies were top priority**<br><br>Studies started faster than usual because everyone was instructed that COVID-19 studies should be top priority. For example, the expert committees that make sure studies respect research ethics put COVID-19 studies at the top of their list to review. Some committees met more often than usual so that they could review studies faster.	**Les études COVID-19 étaient la priorité absolue**<br><br>Les études ont commencé plus rapidement que d'habitude car tout le monde a été informé que les études COVID-19 devaient être prioritaires. Par exemple, les comités d'experts qui veillent à ce que les études respectent l'éthique de la recherche ont placé les études COVID-19 en tête de leur liste d'examen. Certains comités se sont réunis plus souvent que d'habitude afin de pouvoir examiner les études plus rapidement.	
**Having lots of COVID-19 cases made vaccine testing go faster**<br><br>A new vaccine's final, largest test (called a Phase 3 trial) stops once a predetermined number of people in the study get the disease. Then the scientists see whether the people who got the disease had received the vaccine or not. This stopping point happened quickly in the first COVID-19 vaccines approved in Canada because those vaccines were tested in countries with lots of COVID-19 cases. Other vaccines are still being tested in places with fewer COVID-19 cases, so those studies are taking longer to reach the stopping point.	**Le nombre élevé de cas de COVID-19 a accéléré les tests de vaccins**<br><br>Le dernier test d'un nouveau vaccin, le plus important (appelé essai de phase 3), s'arrête lorsqu'un nombre prédéterminé de personnes de l'étude contractent la maladie. Les scientifiques voient alors si les personnes atteintes ont reçu le vaccin ou non. Ce point d'arrêt s'est produit rapidement pour les premiers vaccins COVID-19 approuvés au Canada, car ces vaccins ont été testés dans des pays où il y avait beaucoup de cas de COVID-19. D'autres vaccins sont encore testés dans des pays où il y a moins de cas de COVID-19, de sorte que ces études prennent plus de temps pour atteindre le point d'arrêt.	
**Health Canada reviewed data as it became available**<br><br>Usually, Health Canada (Canada's independent health regulatory agency, not affiliated to any political party) gets a big package of	**Santé Canada a examiné les données au fur et à mesure qu'elles étaient disponibles**<br>Habituellement, Santé Canada (l'agence indépendante de réglementation de la santé au Canada, non affiliée à un parti politique) ne reçoit les résultats	

thousands of pages of scientific results only once all the studies are done. It takes the scientists at Health Canada months to go through it all. For COVID-19 vaccines, they got the data along the way as each step of testing was completed. This “rolling review” meant that once the last set of data came in, the scientists at Health Canada only had to review that last set. The rest of the work was already done.	scientifiques qu'une fois toutes les études terminées. Il faut des mois aux scientifiques de Santé Canada pour tout parcourir. Pour les vaccins COVID-19, ils ont obtenu les données en cours de route, à chaque étape des tests. Cette "révision continue" signifie qu'une fois que la dernière série de données est arrivée, les scientifiques de Santé Canada n'ont eu qu'à examiner cette dernière série. Le reste du travail était déjà fait.	
To make COVID-19 vaccines available in Canada, a lot of the unnecessary waiting and delays were removed. No corners were cut. All the safety checks were still done. All approved COVID-19 vaccines have gone through pre-clinical testing (testing not in humans) and then three phases of clinical studies in humans:<br>- a small (often 10–50 people) Phase 1 trial to make sure there are no major safety issues<br>- a medium (often 20-100 or more people) Phase 2 trial to find out how much of the vaccine to give people and to continue to test its safety<br>- a large (often thousands of people and for COVID-19 vaccines, tens of thousands of people) Phase 3 trial to make sure the vaccine works to prevent disease and to check for rare safety issues	Pour rendre les vaccins COVID-19 disponibles au Canada, une grande partie des attentes et des retards inutiles ont été supprimés. Aucun détail n'a été négligé. Tous les contrôles de sécurité étaient encore effectués. Tous les vaccins COVID-19 approuvés ont fait l'objet de tests pré-cliniques (tests non effectués sur l'homme), puis de trois phases d'études cliniques sur l'homme :<br>- un petit essai de phase 1 (souvent de 10 à 50 personnes) pour s'assurer qu'il n'y a pas de problèmes de sécurité majeurs<br>- un essai de phase 2 de taille moyenne (souvent 20 à 100 personnes ou plus) pour déterminer la quantité de vaccin à administrer et pour continuer à tester son innocuité<br>- un vaste essai de phase 3 (souvent des milliers de personnes et pour les vaccins COVID-19, des dizaines de milliers de personnes) pour s'assurer que le vaccin fonctionne pour prévenir la maladie et vérifier les rares problèmes de sécurité	

When COVID-19 vaccines are approved by Health Canada, it means scientists at Health Canada have carefully checked all the data from all those studies. Once a vaccine is approved, it is no longer experimental. The experiments are done. They showed the vaccines are safe and they work.	Lorsque les vaccins COVID-19 sont approuvés par Santé Canada, cela signifie que les scientifiques de Santé Canada ont soigneusement vérifié toutes les données de ces études. Une fois qu'un vaccin est approuvé, il n'est plus expérimental. Les expériences sont terminées. Elles ont montré que les vaccins sont sûrs et qu'ils fonctionnent.	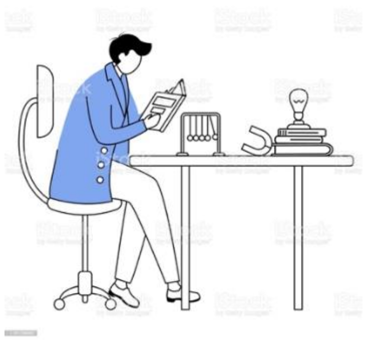
**Version A**<br><br>We are Canadian scientists, doctors, nurses, and experts in health and vaccines. None of us work for pharmaceutical companies. We don't accept pharmaceutical funds either. We will get the COVID-19 vaccines for ourselves as soon as they are approved by Health Canada and available to us. Once they have been tested and approved for children, our children will get the vaccines, too.	**Version A**<br><br>Nous sommes des scientifiques, des médecins, des infirmières et des experts canadiens en matière de santé et de vaccins. Aucun d'entre nous ne travaille pour des sociétés pharmaceutiques. Nous n'acceptons pas non plus de fonds pharmaceutiques. Nous nous ferons vacciner, dès que les vaccins COVID-19 approuvés par Santé Canada seront mis à notre disposition. Une fois qu'ils auront été testés et approuvés pour les enfants, nos enfants recevront eux aussi les vaccins.	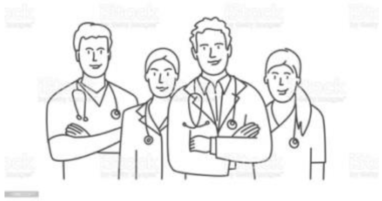
**Version B**<br><br>We are Canadian scientists, doctors, nurses, and experts in health and vaccines. None of us work for pharmaceutical companies. We don't accept pharmaceutical funds either. We will get the COVID-19 vaccines for ourselves as soon as they are approved by Health Canada and available to us. Once they have been tested and approved for children, our children will get the vaccines, too.	**Version B**<br><br>Nous sommes des scientifiques, des médecins, des infirmières et des experts canadiens en matière de santé et de vaccins. Aucun d'entre nous ne travaille pour des sociétés pharmaceutiques. Nous n'acceptons pas non plus de fonds pharmaceutiques. Nous nous ferons vacciner, dès que les vaccins COVID-19 approuvés par Santé Canada seront mis à notre disposition. Une fois qu'ils auront été testés et approuvés pour les enfants, nos enfants recevront eux aussi les vaccins.	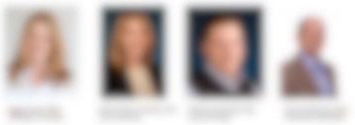
We want to use our scientific training to help answer your questions. Still have questions about COVID-19 vaccines and don't know where to ask? Send us your	Nous voulons utiliser notre formation scientifique pour répondre à vos questions. Vous avez encore des questions sur les vaccins COVID-19 et vous ne savez pas où les poser ?	

question here. We will do our very best to answer as many as we can.	Envoyez-nous votre question ici. Nous ferons de notre mieux pour répondre à autant de questions que possible.	
Thank you!	Merci!	
