# Supplementary material for: Interactive, Personalized Patient Decision Aid for COVID-19 Vaccination in Canada: User-Centered Design Approach
Source: JMIR Hum Factors. 2026 Apr 16;13:e86283. doi: 10.2196/86283 (PMC13086185; doi:10.2196/86283)
Supplement: Multimedia Appendix 5 [file humanfactors-v13-e86283-s005.pdf]

Technical development and version control workflow for VaxDA-C19

Our development team used a structured Git workflow to manage and document the iterative build of VaxDA-C19. We organized the codebase into three main branches: the Dev Branch (249 commits) for implementing new features and experimental changes; the NewDesign Branch (367 commits) for redesigning the user interface based on usability testing feedback; and the Master Branch (308 commits) for stable, production-ready versions. This structure allowed the team to implement updates rapidly, track changes precisely, and maintain proper version histories throughout the project.

We integrated user feedback from each testing cycle directly into the development stream. Developers tagged and documented all modifications related to visual layout, navigation logic, content rendering, and internalization. We relied on JSON-based templates to allow dynamic content updates and easy localization in both English and French.

To ensure reproducibility and transparency, the team mirrored the staging and production environments and followed continuous integration practices to detect bugs early and maintain deployment stability. This technical framework allowed us to integrate public health guidance changes while preserving the accuracy and consistency of the patient decision aid.
