# Supplementary material for: Interactive, Personalized Patient Decision Aid for COVID-19 Vaccination in Canada: User-Centered Design Approach
Source: JMIR Hum Factors. 2026 Apr 16;13:e86283. doi: 10.2196/86283 (PMC13086185; doi:10.2196/86283)
Supplement: Multimedia Appendix 6 [file humanfactors-v13-e86283-s006.pdf]

Qualitative findings from user testing and expert review (S1-S5)

Table S1. Cycle 2 user testing participants' thematic understanding of vaccine explanation text (n=22)

Themes	Feedback	Version A (n=11) n (%)	Version B (n=11) n (%)	Action taken
**Understanding**	Clear explanation of the vaccine development process	7 (64)	9 (82)	Retained detailed version
**Interpretation**	These are the scientists who worked on the vaccines	5 (46)	5 (46)	Clarified scientist roles
**Interpretation**	Text aims to ease anxieties or respond to anti-vaccine concerns	3 (27)	4 (36)	Retained supportive messaging
**Interpretation**	Vaccine research is **not** funded by pharmaceutical companies	2 (18)	2 (18)	Clarified funding explicitly
**Interpretation**	None of the above	1 (9)	1 (9)	

Table S2. Summary of emotional reactions and information gaps

Theme	Key issues	Example quotes	Frequency
**Detachment or suspicion**	Authorities perceived as separate or untrustworthy entities	"The people that made it are vouching for it."<br>"'Them'" trying to explain[…], "in 'their' opinion, [a] vaccine is a source of cure."	2/22
**Information gaps**	Lack of clarity on vaccine effectiveness, side effects, and allergens	"Now it's presented as a miracle that will solve everything."<br>"I'm a bit perplexed."	≥2/22
**Confusion or ambiguity**	Misinterpretation of the statement about vaccine testing locations	"Would need more time to understand this one."<br>"I find the last sentence a little ambiguous."	2/22
**Political or promotional tone**	Perceived political bias and promotional language	"Canada has a ton of vaccines!"	1/22
**Societal consequences emphasis**	Insufficient focus on consequences of non-vaccination	"It's basically a crime if people are not taking this seriously."	1/22
**Perceived contradictions**	Confusion about consistency and accuracy of provided information	"Scientists have been working on the coronavirus since 2003 […] It's not new. [...] Even health ministers from different provinces say different things."	1/22

Table S3. Themes of unanswered questions (n=22)

Theme	Feedback	n (%)
General mechanisms and effects	How does the vaccine work? What does it do to the virus? Why two doses? What does it do to your body? Are there side effects?	9 (41)
COVID-19 vaccine-specific information	What is this technology? Why are there different vaccines? How were they made, what's in them? What are variants and do the vaccines cover them? What protection does it give? How long before it kicks in? If I get COVID-19, does it mean I don't need a vaccine? Is it like the flu vaccine that you should get each year?	12 (55)
Distribution and eligibility	How are they distributed, when can we get it? Who should and shouldn't get the vaccine? What happens if you don't get the vaccine?	7 (32)
Safety and research	Is the vaccine safe? Were they tested enough? Is there enough data? Were people with different conditions part of the trials?	6 (27)
No remaining questions	-	2 (9)
Question not asked	-	1 (5)

Table S4. Summary of thematic feedback from Cycle 3 user testing

Pages	Themes	Feedback
Home Page<br>Display Text/Instructions (1.0)	First reaction	- Good and draws me to search for more<br>- Confusing header<br>- Informative, supports evidence-informed decisions
	Understanding	- Facilitates COVID-19 vaccine decision-making
	Confusion	- Unclear header<br>- Difficulty finding "Next" button
	Suggestions	- Clarify header to indicate vaccine comparison explicitly<br>- Differentiate content for vaccinated/unvaccinated users
Choices<br>Sociodemographic and vaccine data entry (2.0)	First reaction	- Find a comparison of vaccines due to controversies<br>- Interesting
	Understanding	- Comparing efficacy for different age groups<br>- Variation in dosage and learning more<br>- Getting data on vaccines
	Confusion	- Buttons not showing expected comparisons<br>- Expected information on side effects and efficacy not displayed<br>- Comparing with "no vaccine" perceived unhelpful<br>- Technical issue with unresponsive "Next" button
	Suggestions	- Provide information on efficacy and side effects when selecting vaccines<br>- Ensure transparency, especially for younger age groups<br>- Explicitly indicate information sources
Avatar<br>Build an avatar (3.0)	First reaction	- Curious about the following information<br>- Relevant if having a child
	Understanding	- Relevant for child-related decisions
	Confusion	- Avatar creation perceived as irrelevant or time-consuming
	Suggestions	- Provide option to bypass avatar creation
Compare<br>Display evidence of risks and benefits (4.0)	First reaction	- Comparison of vaccine types with evidence
	Understanding	- Explains and compares each vaccine side by side
	Confusion	- Unclear grid lines for comparison
	Suggestions	- Improve grid visibility
Community<br>Display evidence of population benefits (5.0)	First reaction	- Prefers factual text over animations
	Understanding	- Explains herd immunity, but more text needed
	Confusion	- Prefers direct information rather than interactive elements like avatar selection
	Suggestions	- Provide direct textual information
Consider<br>Values Clarification Sliders (6.0)	First reaction	- Confusion regarding slider functionality
	Understanding	- Supports vaccine choice based on personal values
	Confusion	- Slider interactions unclear<br>- Slider colors are dull<br>- Unclear medical terminology
	Suggestions	- Clarify slider instructions<br>- Enhance slider color contrast

		- Clearly define medical terms
FAQ<br>Frequently asked questions<br>(7.0)	First reaction	- Quick way to get answers to common questions
	Understanding	- Information on commonly asked questions about vaccines
	Confusion	- None identified
Sources<br>About us (8.0)	First reaction	- Actionable information on testing/vaccine locations
	Understanding	- Provides outbreak updates and location information
	Confusion	- None identified

Table S5. Cycle 4: Expert review feedback and implemented modifications

Section	Expert feedback	Priority	Implemented modifications
**Avatar creation**	Improve logical flow	High	Repositioned avatar creation
**Decision clarity**	Clarify vaccine selection instructions	High	Added explicit instructional texts
**Decision clarity**	Include the "I don't know" option clearly	High	Option clearly added
**Mobile interface**	Resolve text overlap and navigation issues	High	Improved responsive design and optimized mobile usability
**Language**	Complete bilingual implementation	High	Fully implemented bilingual interface (English/French)
**Content clarity**	Clarify wording of decision context (e.g., "I am deciding about…")	High	Decision wording clarified consistently across all steps
**Community immunity animation**	Adjust slow-running animations, clarify instructions, add a skip function	High	Animation optimized, explicit instructions provided, skip function added
**Values clarificatior sliders**	Address confusion around slider instructions and colors	High	Instructions repositioned clearly above sliders, colors adjusted for clarity
**Avatar usability**	Clarify the avatar skipping process	High	Clear option added to bypass avatar creation easily
**Navigation**	Allow free navigation across sections via the bottom path bar	Medium	Navigation bar updated for easier movement between sections
**Home page**	Display funder/institutional logos and contact information	Medium	Logos and contact details added clearly on the homepage and FAQ
**Visual layout**	Improve spacing of provincial selection buttons	Low	Implemented in the final version
**Icon arrays**	Resolve color confusion with face icons	Low	Not implemented (consideration for future visual redesign)
**Community immunity section**	Add customizable animation speed/pause options	Low	Not implemented (evaluated for technical complexity in future versions)
**Information densit**	Reduce textual density	Low	Not implemented (noted for future text optimization)
**Vaccine comparison**	Simplify side-by-side visual vaccine comparison visuals difficult	Low	Not implemented (requires future technical exploration)
