# Supplementary material for: Interactive, Personalized Patient Decision Aid for COVID-19 Vaccination in Canada: User-Centered Design Approach
Source: JMIR Hum Factors. 2026 Apr 16;13:e86283. doi: 10.2196/86283 (PMC13086185; doi:10.2196/86283)
Supplement: Checklist 1 [file humanfactors-v13-e86283-s007.pdf]

DEVELOPTOOLS Reporting Checklist

Legend: ✓ Yes; ◐ Partially; ✗ Not addressed; — not applicable

Factor: Preprototype involvement		
✓	1. Were potential users (patients, caregivers, family and friends, surrogates) involved in any steps to help understand users (e.g., who they are, in what context might they use the tool) and their needs?	**Yes.** We involved potential end users early to understand who they are, how they might use the patient decision aid, and what they need. In cycle 1, we recruited nine adults in Canada through tailored Facebook ads and ran individual semi-structured Zoom interviews with screen sharing. We asked participants to summarize what they understood, point out confusing parts or unanswered questions, and react to wording. We also used comparative testing in Cycle 1 (for example, two icon array styles) to learn how people understood risk information and what they preferred. In parallel, a citizen panel (n=6) met throughout the project and gave ongoing advice on the design and development of the patient decision aid and our user testing methods and results.<br><br>**Number reported**<br>• Potential end users (total): n = 34 | Cycle 1 (n = 9), Cycle 2 (n = 22), Cycle 3 (n = 3)<br>• Citizen panel (advisory): n = 6 | ongoing across cycles (separate from user testing)
✓	2. Were potential users (patients, caregivers, family and friends, surrogates) involved in any steps of designing, developing, and/or refining a prototype?	**Yes.** Potential end users helped us design, develop, and refine prototypes across the early and the middle phases of development. In Cycle 1, we built an initial content mock-up and asked nine adults in Canada to review draft content and visuals, identify unclear parts, and suggest changes. In Cycle 2, we created higher-fidelity Google Slides mockups that represented a proposed website and user flow. We then asked 22 new adults in Canada to review these mock-ups, compare alternative design and content presentations (comparative testing), and tell us what to change before we invested in a complete web development. We used their feedback to refine wording, structure, and interface elements (e.g, the user flow and how we presented information) and to guide subsequent feature development.<br><br>**Number reported**<br>• Potential end users (total): n = 34 | Cycle 1 (n = 9), Cycle 2 (n = 22), Cycle 3 (n = 3)<br>• Citizen panel (advisory): n = 6 | ongoing across cycles (separate from user testing)

Factor: Iterative responsiveness		
✓	3. Were potential users (patients, caregivers, family and friends, surrogates) involved in any steps intended to evaluate prototypes of the tool or a final version of the tool?	**Yes.** Potential end users evaluated prototypes in multiple rounds of user testing. We conducted remote usability testing with adults in Canada via Zoom and progressively higher-fidelity prototypes across iterative cycles. In cycles 1 and 2, we conducted individual semi-structured interviews to evaluate early prototypes (a content mock-up, then website mock-ups) and identify usability issues, misunderstandings, and missing information. In cycle 3, we moved from a static mock-up to a functional web prototype. We asked new adult participants to test it using think-aloud protocols, focusing on navigation, multiple device compatibility, and overall usability. We used findings from each cycle to guide revisions before moving to the next version.<br><br>**Number reported**<br>● Potential end users (total): n = 34 | Cycle 1 (n = 9), Cycle 2 (n = 22), Cycle 3 (n = 3)
✓	4. Were potential users (patients, caregivers, family and friends, surrogates) asked their opinions of prototypes of the tool or a final version of the tool in any way?	**Yes.** We asked potential end users for their opinion on VaxDA-C19 prototypes in every user testing cycle. In Cycle 1 and 2, we asked participants to explain what they understood, highlight confusing parts, and tell us what felt unclear or missing. We also used comparative testing to capture preferences and reactions to alternative designs and content presentation (e.g., icon-array styles and team presentation formats). In Cycle 3, we asked participants to test the functional web prototype using think-aloud protocols and to comment on navigation, clarity, and overall usability.<br><br>**Number reported**<br>● Potential end users (total): n = 34 | Cycle 1 (n = 9), Cycle 2 (n = 22), Cycle 3 (n = 3)
✓	5. Were potential users (patients, caregivers, family and friends, surrogates) observed using the tool in any way?	**Yes.** We observed potential end users during remote user testing on Zoom. In Cycles 1 and 2, the interviewer shared the screen to show the mock-ups and watched participants' reactions while they interpreted the content and visuals, pointed out confusing parts, and described what they would click or read next. In Cycle 3, participants shared their own screen and used the functional web prototype while thinking aloud. We observed how they moved through the user flow, navigated the pages, and interacted with key features.<br><br>**Number reported**<br>● Potential end users (total): n = 34 | Cycle 1 (n = 9), Cycle 2 (n = 22), Cycle 3 (n = 3)

✓	6. Did the development process have 3 or more iterative cycles?	**Yes.** We ran four iterative development cycles. After each cycle, we reviewed feedback from external stakeholders and made changes before moving to the next version. Cycles 1 and 2 focused on early content and website mock-ups, Cycle 3 tested a functional web prototype, and Cycle 4 focused on expert review and final refinements.<br><br>**Number of cycles reported**<br>● 4 cycles
✓	7. Were changes between iterative cycles explicitly reported in any way?	**Yes.** We explicitly reported the main changes made between cycles in the manuscript. We described the changes made after each cycle in the cycle descriptions (Cycles 1- 4) and summarized cycle objectives, methods, sample sizes, and key modifications in Table 1 (Cycle Summaries). We also reported specific design and content decisions in the text (e.g., adding interactive features in Cycle 3, restructuring the tool to focus on vaccination decisions, and implementing content and interface refinements after expert review in Cycle 4).<br><br>**What did we do?**<br>● We documented cycle-by-cycle modifications in the narrative (Cycles 1–4)<br>● We summarized key changes in Table 1, including which features were added or revised after each cycle<br>● We described the rationale for major changes based on user and expert feedback (e.g., interface/navigation refinements, adding/adjusting interactive elements, and updating content to match evolving recommendations)
**Factor: Other expert involvement**		
✓	8. Were health professionals asked their opinion of the tool at any point?	**Yes.** We asked health professionals to review the patient decision aid and give feedback during the development process. Health professionals (along with other scientific experts) reviewed the content for scientific accuracy, clarity, and clinical relevance, and they also commented on user experience and navigation. This happened in particular during:<br>● Cycle 3, when experts provided feedback on medical content and interface navigation during a structured discussion after the prototype presentation.<br>● Cycle 4, when experts completed a detailed review of the near-final version, focusing on scientific accuracy, linguistic clarity, completeness, and the overall user journey across the four vaccination decisions.

		**Number reported**<br>• Health professionals are included within: Experts (total): n = 15 | Cycle 3 (n = 6), Cycle 4 (n = 9)<br><br>**What did we do?**<br>• We requested structured feedback from health professionals on the accuracy, clarity, and completeness of the content<br>• We asked them to identify missing or unclear information and to flag any issues that could affect real-world use<br>• We used their feedback to prioritize revisions (e.g., clarifying instructions, removing irrelevant options for some age groups, improving navigation and mobile layout, updating content to reflect current recommendations)
✗	9. Were health professionals consulted before a first prototype was developed?	**No.** Only health professionals who were members of the team were consulted before the first prototype was developed.
✓	10. Were health professionals consulted between initial and final prototypes?	**Yes.** We consulted health professionals after we created initial prototypes and as we moved toward the final version. We built early prototypes (Cycle 1 content mock-up; Cycle 2 website mock-ups) and then sought input from health professionals and other experts as we refined and finalized the tool. In Cycle 3, we tested a functional web prototype and collected expert feedback on content and navigation. In Cycle 4, we asked experts (i.e, co-investigators, including health professionals) to review the near-final version for scientific accuracy, clarity, completeness, and the full user journey across the four vaccination decisions, and we used this feedback to prioritize revisions.<br><br>**Number reported**<br>Experts (total): n = 15 | Cycle 3 (n = 6), Cycle 4 (n = 9)
✓	11. Was an expert panel involved?	**Yes.** Both a citizen panel and outside experts were involved at various points of the development process.
**Additional elements in DEVELOPTOOLS Reporting Checklist**		
✓	12. Was a formal advisory panel of users involved?	**Yes.** We had a citizen panel throughout the project.
✓	13. Were users (patients, caregivers, family and friends, surrogates), health professionals, and other relevant stakeholders involved as members of the	**Yes.** All were involved throughout the project, including as co-authors of papers.

	research team?	
✓	14. Were members of populations marginalized by social norms and policies involved?	**Yes.** Such persons were part of the citizen panel.
✓	15. How many users (patients, caregivers, family and friends, surrogates) and health professionals were involved in total and of each type?	**Yes.** We involved 34 potential end users in user testing across three cycles (Cycle 1: n = 9, Cycle 2: n = 22, Cycle 3: n = 3) and a citizen panel advisory group that met throughout the project (n = 6; separate from user testing). We also obtained expert input from 15 external experts across two cycles (Cycle 3 n = 6, Cycle 4 n = 9), including healthcare professionals, medical decision-making researchers, and user experience specialists. We did not report a separate count for health professionals versus other expert profiles.
✓	16. Does the tool have a defined purpose?	**Yes.** The tool's purpose is to support people in Canada in making evidence-informed, values-congruent decisions about COVID-19 vaccination.
✓	17. Is the tool intended to be used in a particular context?	**Yes.** The tool is intended to be used outside of clinical encounters, as this is where people in Canada often make decisions about vaccination. In other words, because people in Canada may not seek medical advice about annual vaccines, the tool is intended to reach people where they are.
✓	18. Were any methods used to facilitate sharing of perspectives between groups?	**Yes.** We brought insights from the citizen panel to scientific meetings, and vice versa.
✓	19. Were users (patients, caregivers, family and friends, surrogates) involved from the outset of the project?	**Yes.** We established the citizen panel before beginning to develop the tool.
✓	20. Were translation and cultural adaptation used to render the patient decision aid available to users across languages and cultures?	**Yes.** The tool is available in both English and French. Additionally, the tool includes customizable avatars with a range of skin tones, hair styles and cultural head coverings. These features were created in a previous project in response to comments from users from cultures that were not yet represented in the visualization.
